# Long-term effects of SGLT2 inhibitors on arrhythmias: a systematic review and meta-analysis

**DOI:** 10.3389/fphar.2025.1558367

**Published:** 2025-07-02

**Authors:** Peipei Li, Weiwei Chen, Rui Chen, Hanmo Zhang, Zhixi Yu, Hongyu Yan, Beibei Du, Ping Yang

**Affiliations:** ^1^ Department of Cardiology, Jilin Provincial Cardiovascular Research Institute, China-Japan Union Hospital of Jilin University, Changchun, China; ^2^ Cardiovascular Hospital, The First Affiliated Hospital of Zhengzhou University, Zhengzhou University, Zhengzhou, China; ^3^ School of Medicine, Nankai University, Tianjin, China

**Keywords:** sodium-glucose co-transporter 2 inhibitors, arrhythmia, atrial fibrillation, atrial flutter, meta-analysis

## Abstract

**Aims:**

Sodium-glucose co-transporter 2 (SGLT2) inhibitors are novel oral hypoglycemic agents strongly endorsed in the treatment guidelines for heart failure due to their cardioprotective benefits. However, their specific impact of SGLT2 inhibitors on arrhythmias incompletely understood. This systematic review and meta-analysis aimed to comprehensively evaluate the long-term effects of SGLT2 inhibitors on various arrhythmia types.

**Methods:**

We systematically searched PubMed, Embase, Web of Science, and ClinicalTrials.gov from database inception to 30 June 2024, to identify randomized controlled clinical trials (RCTs) with a follow-up duration of at least 52 weeks. The primary outcome of the meta-analysis was atrial fibrillation (AF) or atrial flutter (AFL), and the secondary outcomes included ventricular tachycardia (VT), ventricular fibrillation (VF), and sinus bradycardia. The pooled risk ratios (RRs) with 95% confidence intervals (CIs) were used to estimate the incidence of arrhythmias.

**Results:**

Thirty-nine RCTs involving 107,770 participants were included. The results of meta-analysis revealed that patients treated with SGLT2 inhibitors had a reduced risk of AF/AFL compared with placebo (RR 0.86; 95%CI, 0.77–0.95; I^2^ = 0%; P = 0.003). There was no significant difference in the risk of AF/AFL between the high-dose SGLT2 inhibitors group and the low-dose SGLT2 inhibitors group (RR 0.78; 95%CI, 0.60–1.02; I^2^ = 0%; P = 0.07), although a decreasing trend in the high-dose group was noted. Similarly, no significant differences were found for VT (RR 0.99; 95%CI, 0.81–1.22; I^2^ = 0%; P = 0.96), VF (RR 1.06; 95%CI, 0.73–1.54; I^2^ = 0%; P = 0.75) or sinus bradycardia (RR 1.12; 95%CI, 0.57–2.18; I^2^ = 0%; P = 0.74) between the SGLT2 inhibitors and placebo groups.

**Conclusion:**

SGLT2 inhibitors significantly reduce the risk of AF/AFL but have no notable impact on the risk of VT, VF, and sinus bradycardia. Additionally, different doses of SGLT2 inhibitors did not statistically influence AF/AFL incidence.

**Systematic Review Registration:**

https://www.crd.york.ac.uk/PROSPERO/home, identifier PROSPERO:CRD42022371089

## 1 Introduction

Sodium-glucose co-transporter 2 (SGLT2) inhibitors are novel oral hypoglycemic agents, whose cardioprotective effects have been explored extensively in recent years (Wei and Du, 2023). The 2021 ESC Guidelines for the Management and Treatment of Acute and Chronic Heart Failure recommended SGLT2 inhibitors for patients with type 2 diabetes mellitus (T2DM) at risk of cardiovascular (CV) events, citing their ability to reduce heart failure (HF) hospitalization, major CV events, and CV death. In the absence of contraindications and when tolerated, dapagliflozin or empagliflozin is endorsed for patients with HFrEF, regardless of diabetes status (Mcdonagh et al., 2022). More recently, SGLT2 inhibitors have been recommended for the management of heart failure with mildly reduced (HFmrEF) or preserved ejection fraction (HFpEF) (class IIA), according to the 2022 AHA/ACC/HFSA Heart Failure Management Guidelines. These drugs have demonstrated benefits in reducing the rehospitalization rate and CV mortality in HFmrEF and HFpEF patients (Heidenreich et al., 2022). Additionally, animal studies have highlighted the potential of SGLT2 inhibitors in mitigating atherosclerosis progression (Ganbaatar et al., 2020; Al-Sharea et al., 2018; Nasiri-Ansari et al., 2018; Nakatsu et al., 2017).

The 2024 ESC Guidelines for the management of atrial fibrillation recommend effective glycemic control as part of comprehensive risk factor management in individuals with diabetes mellitus and AF. This approach is beneficial for reducing burden, recurrence, and progression of AF (class IC) (Van Gelder et al., 2024). As novel hypoglycemic agents with cardiovascular benefits, SGLT2 inhibitors have been shown to have potential antiarrhythmic effects in limited clinical and animal researches. In a *post hoc* analysis of DECLARE-TIMI58, Zelniker et al. (2020) observed that dapagliflozin significantly reduced the incidence of atrial fibrillation (AF) and atrial flutter (AFL) in patients with T2DM, irrespective of prior history of AF, atherosclerotic heart disease or HF. Our previous research further demonstrated the anti-arrhythmic potential of empagliflozin, in an *ex-vivo* myocardial ischemia-reperfusion rabbit model (Azam et al., 2021). Despite several meta-analyses evaluating the effects of SGLT2 inhibitors on AF/AFL, their findings remain inconsistent (Li et al., 2021; Pandey et al., 2021; Zhang et al., 2024). The association between SGLT2 inhibitors and the other arrythmias remains even less explored. To address this gap, we performed a systematic review and meta-analysis of randomized controlled clinical trials (RCTs) evaluating the impact of SGLT2 inhibitors on various arrhythmias, including AF/AFL, ventricular tachycardia (VT), ventricular fibrillation (VF), and sinus bradycardia, with the ultimate goal of informing evidence-based clinical decision-making.

## 2 Methods

This research strictly adhered to the Guidelines for Systematic Review and Meta-Analysis (PRISMA) statement (Page et al., 2021) across all stage, including data sources, search strategies, data acquisition, inclusion and exclusion criteria, outcome measures, quality assessment, and statistical methods. The protocol for this systematic review and meta-analysis was registered with the International Prospective Register of Systematic Reviews (PROSPERO: CRD42022371089).

### 2.1 Inclusion and exclusion criteria

The inclusion and exclusion criteria were developed based on PICOTS (Table 1).

**TABLE 1:** Inclusion criteria and exclusion criteria.

PICOTS	Inclusion criteria	Exclusion criteria
Participant (P)	Adults aged 18 years or older	
Intervention (I)	SGLT2 inhibitors (dapagliflozin, empagliflozin, canagliflozin, ertugliflozin, tofogliflozin, luseogliflozin, ipragliflozin, remogliflozin and sergliflozin) and SGLT1/2 inhibitors (sotagliflozin and licogliflozin)	
Control (C)	Placebo	
Outcome (O)	AF, AFL, VT, VF, and sinus bradycardia	
Time (T)	Follow-up duration ≥ 52 weeks	
Study (S)	RCTs	Non-randomized controlled trials, animal studies, reviews, meta-analyses, case reports, reviews, abstracts of meetings, letters, guidelines, expert consensuses, and non-English literatures were excluded

The inclusion criteria included: (1) Adult participants (≥18 years old); (2) Intervention group treated with SGLT2 inhibitors (dapagliflozin, empagliflozin, canagliflozin, ertugliflozin, tofogliflozin, luseogliflozin, ipragliflozin, remogliflozin and sergliflozin) or SGLT1/2 inhibitors (sotagliflozin and licogliflozin), and control group (placebo); (3) Follow-up duration≥52 weeks; (4) Report of arrhythmia events (AF, AFL, VT, VF. and sinus bradycardia); (5) RCTs.

Exclusion criteria encompassed non-randomized placebo-controlled trials, animal studies, reviews, meta-analyses, case reports, letters, guidelines, expert consensuses, and non-English literatures.

### 2.2 Search strategy and data sources

We systematically searched of PubMed, Embase, Web of Science, and ClinicalTrials.gov for relevant studies published from each database’s inception up to 30 June 2024. The search term in ClinicalTrials.gov was “Sodium-Glucose Transporter 2 Inhibitors” with the filters set as “with results,” “intervention studies,” “adults (18–64),” and “older adults (65+).” The search terms in PubMed, Embase, Web of Science included “sodium glucose cotransporter 2 inhibitor,” “dapagliflozin,” “empagliflozin,” “canagliflozin,” “tofogliflozin,” “luseogliflozin,” “ertugliflozin,” “sergliflozin,” “ipragliflozin,” “remogliflozin,” “sotagliflozin,” “licogliflozin,” “atrial fibrillation,” “atrial flutter,” “tachycardia, ventricular,” “ventricular fibrillation,” “sinus bradycardia” and other relevant terms. Specific search strategies were detailed in the Supplementary Table S1.

### 2.3 Study selection, data extraction and quality assessment

All the studies were independently identified, reviewed, and screened by two reviewers (Z.X. Yu and H.Y. Yan) based on titles, abstracts and full texts. Disagreements were resolved through discussion or third-party consultation (W.W. Chen).

Using a unified data extraction form, two reviewers (R. Chen and H.M. Zhang) independently abstracted data on intervention and outcome, and recorded study and participant characteristics. Disagreements were resolved through discussion or third-party consultation (P.P. Li).

The risk of bias was assessed using the domains suggested in the Cochrane Handbook for Systematic Review of Interventions, Version 5.1.0, (Higgins JPT, Green S, eds. Cochrane Handbook for Systematic Reviews of Interventions [version 5.1.0. updated March 2011] http://handbook.Cochrane.org/. Accessed 6 August 2024), including selection bias (random sequence generation, allocation concealment), performance bias (blinding of participants and personnel), detection bias (blinding of outcome assessment), attrition bias (incomplete outcome data), reporting bias (selective reporting) and other bias.

### 2.4 Data analysis

The primary outcome was the incidence of AF/AFL, while the secondary outcomes comprised VT, VF, and sinus bradycardia.

The pooled risk ratios (RRs) with 95% confidence intervals (CIs) were used to estimate incidence of arrhythmias, with P-values <0.05 considered statistical significance. Heterogeneity was assessed using Q tests and I^2^ statistics. The Mantel–Haenszel test with fixed-effects model was applied when P-value for Q test>0.1 and I^2^<50%, while random-effects model was used otherwise. Funnel plots were used to evaluate the publication bias of included studies. All analyses followed the intention-to-treat principle and were conducted using RevMan 5.4.1 (The Cochrane Collaboration). The sensitivity analysis was conducted using the leave-one-out method to evaluate the reliability of the results.

## 3 Results

### 3.1 Screening

**FIGURE 1:**
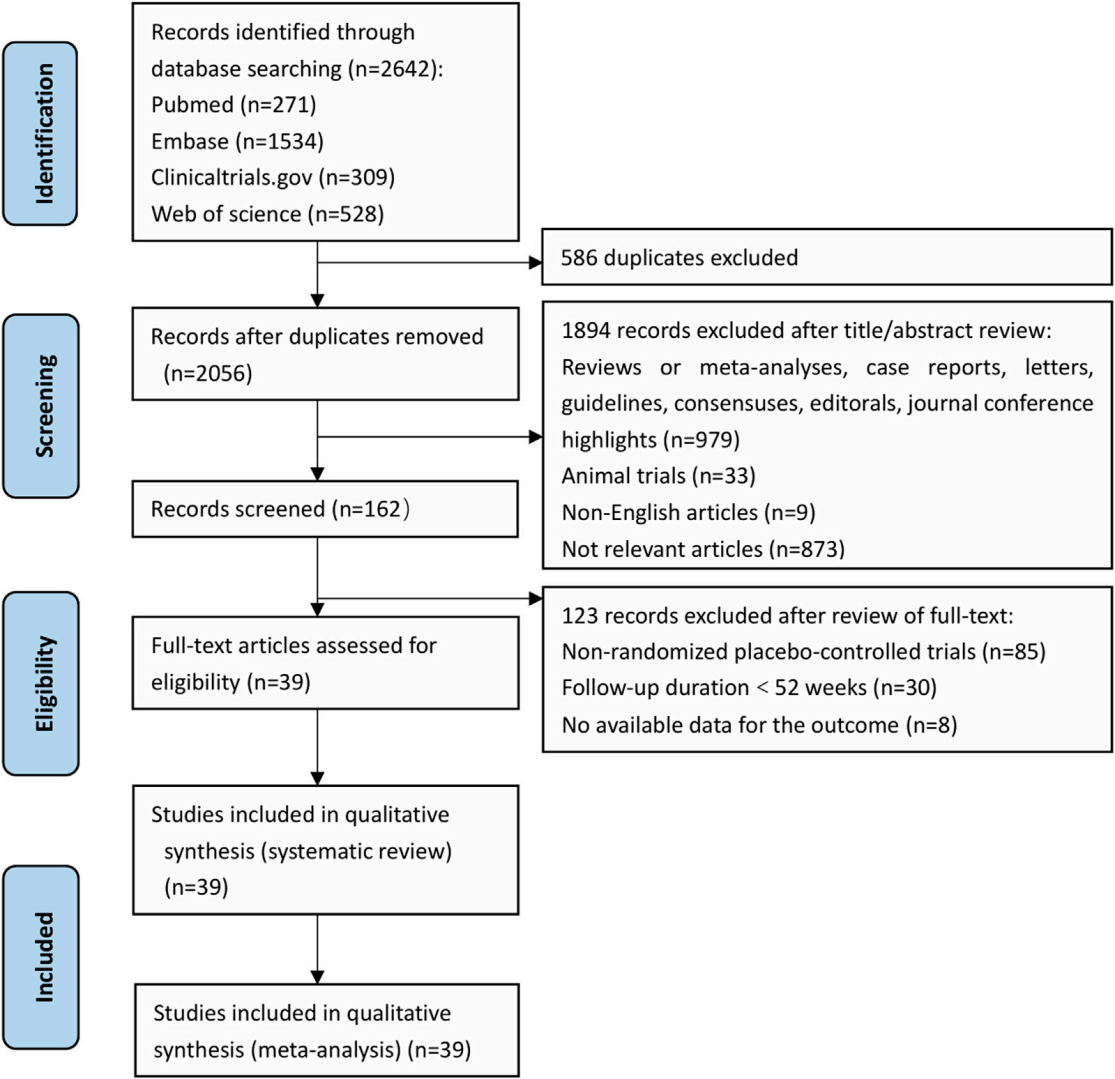
PRISMA Systematic Review Flow Diagram. PRISMA, Preferred Reporting Items for Systematic Reviews and Meta-analyses.

A total of 2,642 relevant studies were identified initially, and 39 RCTs were included after screening. The screening process was illustrated in Figure 1.

### 3.2 Baseline characteristics of included studies and bias risk assessment

**FIGURE 2:**
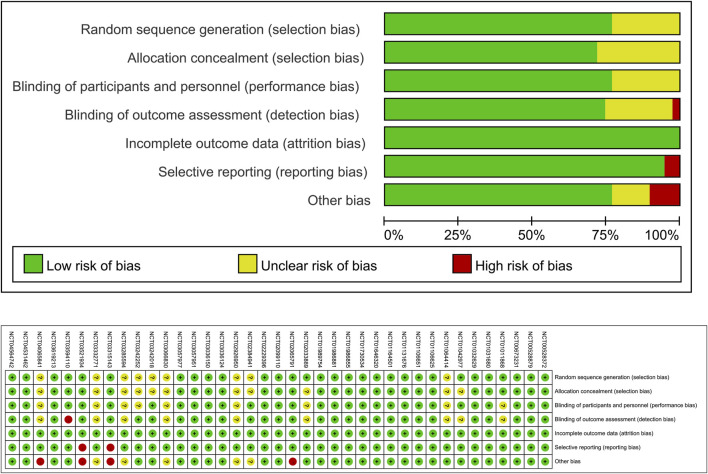
Quality assessment of included RCTs.

39 RCTs (Bode et al., 2013; Neal et al., 2017; Perkovic et al., 2019; Wildi et al., 2013; Yale et al., 2014; Bailey et al., 2013; Bailey et al., 2015; Cefalu et al., 2015; Heerspink et al., 2020; Jabbour et al., 2020; Leiter et al., 2014; Mathieu et al., 2015; Mcmurray et al., 2019; Wilding et al., 2014; Wiviott et al., 2019; Anker et al., 2021; Barnett et al., 2014; Packer et al., 2020; Rosenstock et al., 2015; Zinman et al., 2015; Cannon et al., 2020; Grunberger et al., 2018; Pratley et al., 2018; Rosenstock et al., 2018; Bhatt et al., 2021a; Bhatt et al., 2021b; Buse et al., 2018; Cher et al., 2021; Cherney et al., 2023; Clinical trail, 2021c; Clinical trail, 2021a; Clinical trail, 2021d; Clinical trail, 2021b; Herrington et al., 2023; James et al., 2024; Mc Causland et al., 2023; Yabe et al., 2021; Clinical trail, 2024) were included in this meta-analysis. These trials assessed various SGLT2 inhibitors: dapagliflozin (12 trials), empagliflozin (7 trials), canagliflozin (6 trials), sotagliflozin (9 trials), ertugliflozin (4 trials), and licogliflozin (1 trial), encompassing a total of 107,770 participants. Table 2 summarizes the baseline characteristics of the included RCTs. The quality of the trials was assessed using the Cochrane Risk of Bias Tool, with results shown in Figure 2.

**TABLE 2:** Baseline characteristics of included RCTs.

NCT number	Author, Year	Acronym	Simple size	Population	Age (mean ± SD)		Female ratio (%)		Intervention	Follow-up duration
					SGLT2i	Control	SGLT2i	Control		
NCT04564742	James et al. (2024)	DAPA-MI	4,017	AMI	63.0 ± 11.06	62.8 ± 10.64	19.2	21	Dapagliflozin 10 mg	12 months
NCT03619213	Mc Causland et al. (2023)	DELIVER	6,263	EFpHF	71.8 ± 9.6	71.5 ± 9.5	43.6	44.2	Dapagliflozin 10 mg	42.2 months
NCT03036150	Heerspink et al. (2020)	DAPA-CKD	4,304	CKD	61.8 ± 12.1	61.9 ± 12.1	32.9	33.3	Dapagliflozin 10 mg	39.2 months
NCT02229396	Jabbour et al. (2020)	DURATION-8	695	T2D	53.8 ± 9.8	54.2 ± 9.6	55.3	48.9	Dapagliflozin 10 mg	104 weeks
NCT03036124	McMurray et al. (2019)	DAPA-HF	4,744	EFrHF	66.2 ± 11.0	66.5 ± 10.8	23.8	23	Dapagliflozin 10 mg	28.3 months
NCT01730534	Wiviott et al. (2019)	DECLARE-TIMI58	17,160	T2D, High Risk for Cardiovascular Event	63.9 ± 6.8	64 ± 6.8	36.9	37.9	Dapagliflozin 10 mg	5.2 years
NCT01646320	Mathieu et al. (2015)	——	320	T2D	55.2 ± 8.6	55 ± 9.6	56.3	52.5	Dapagliflozin 10 mg	52 weeks
NCT00528372	Bailey et al. (2015)	——	558	T2D	52.6 ± 10.8	52.7 ± 10.3	51.7	58.7	Dapagliflozin 2.5 mg, 5 mg and 10 mg	102 weeks
NCT01031680	Cefalu et al. (2015)	——	922	T2D, CVD, Hypertension	62.8 ± 7.0	63 ± 7.7	32.1	31.4	Dapagliflozin 10 mg	52 weeks
NCT01042977	Leiter et al. (2014)	——	965	T2D, CVD	63.9 ± 7.6	63.6 ± 7.0	33.1	33	Dapagliflozin 10 mg	52 weeks
NCT00673231	Wilding et al. (2014)	——	807	T2D	59.5 ± 8.1	58.8 ± 8.6	52.7	50.8	Dapagliflozin 2.5 mg, 5 mg and 10 mg	80 weeks
NCT00528879	Bailey et al. (2013)	——	546	T2D	54.0 ± 9.6	53.7 ± 10.3	47.2	44.5	Dapagliflozin 2.5 mg, 5 mg and 10 mg	102 weeks
NCT03594110	Herrington et al. (2023)	EMPA-KIDNEY	6,609	CKD	63.4 ± 13.9	63.3 ± 13.9	33.2	33.1	Empagliflozin 10 mg	1,147 days
NCT04531462	Yabe et al. (2021)	EMPA-ELDERLY	129	T2D	74.2 ± 4.9	74.0 ± 5.1	25.00	30.20	Empagliflozin 10 mg	52 weeks
NCT03057951	Anker et al. (2021)	EMPEROR-Preserved	5,988	EFpHF	71.8 ± 9.3	71.9 ± 9.6	44.6	44.7	Empagliflozin 10 mg	1,403 days
NCT03057977	Packer et al. (2020)	EMPEROR-Reduced	3,730	EFrHF	67.2 ± 10.8	66.5 ± 11.2	23.5	24.4	Empagliflozin 10 mg	1,040 days
NCT01131676	Zinman et al. (2015)	EMPA-REG OUTCOME	7,028	T2D, High Risk for Cardiovascular Event	63.1 ± 8.6	63.2 ± 8.8	28.8	28	Empagliflozin 10 mg and 25 mg	5 years
NCT01011868	Rosenstock et al. (2015)	——	494	T2D	59.2 ± 10.1	58.1 ± 9.4	42.6	47.1	Empagliflozin 10 mg and 25 mg	82 weeks
NCT01164501	Barnett et al. (2014)	——	741	T2D, CKD	63.7 ± 8.9	64.1 ± 8.7	40.6	43.3	Empagliflozin 10 mg and 25 mg	458 days
NCT02065791	Perkovic et al. (2019)	CREDENCE	4,401	T2D, CKD	62.9 ± 9.2	63.2 ± 9.2	34.6	33.3	Canagliflozin 100 mg	4.6 years
NCT01032629	Neal et al. (2017)	CANVAS	4,330	T2D, High Risk for Cardiovascular Event	62.5 ± 8.1	62.3 ± 7.9	34	33.7	Canagliflozin 100 mg and 300 mg	8 years
NCT01989754	Neal et al. (2017)	CANVAS-R	5,812	T2D, High Risk for Cardiovascular Event	63.9 ± 8.4	64 ± 8.23	36.2	38.2	Canagliflozin from 100 mg to 300 mg	3 years
NCT01064414	Yale et al. (2014)	——	272	T2D, Renal Insufficiency	68.7 ± 8.2	68.2 ± 8.4	40.8	36.7	Canagliflozin 100 mg and 300 mg	52 weeks
NCT01106651	Bode et al. (2013)	——	716	T2D	63.9 ± 6.2	63.2 ± 6.2	47	39.7	Canagliflozin 100 mg and 300 mg	104 weeks
NCT01106625	Wilding et al. (2013)	CANTATA-MSU	469	T2D	56.7 ± 9.8	56.7 ± 8.4	47.9	51.3	Canagliflozin 100 mg and 300 mg	52 weeks
NCT01986881	Cannon et al. (2020)	VERTIS CV	8,246	T2D, Atherosclerosis (AS)	64.4 ± 8.1	64.4 ± 8.0	29.7	30.7	Ertugliflozin 5 mg and 15 mg	6 years
NCT02033889	Rosenstock et al. (2018)	VERTIS MET	621	T2D	56.7 ± 8.8	56.5 ± 8.7	53.9	53.1	Ertugliflozin 5 mg and 15 mg	106 weeks
NCT01986855	Grunberger et al. (2018)	VERTIS RENAL	468	T2D, CKD	67.1 ± 8.4	67.5 ± 8.9	49.2	53.2	Ertugliflozin 5 mg and 15 mg	54 weeks
NCT02099110	Pratley et al. (2018)	VERTIS FACTORAL	1,233	T2D	55.1 ± 10.1	54.8 ± 10.7	48.9	37.7	Ertugliflozin 5 mg and 15 mg	54 weeks
NCT03242252	Cherney et al. (2023)	SOTA-CKD3	787	T2D, CKD Stage 3	69.5 ± 7.9	69.3 ± 8.1	44	42.7	Sotagliflozin 200 mg and 400 mg	60 weeks
NCT03242018	Cherney et al. (2021)	SOTA-CKD4	277	T2D, CKD Stage 4	67.1 ± 9.8	68.0 ± 8.3	49.5	54.8	Sotagliflozin 200 mg and 400 mg	60.3 weeks
NCT03521934	Bhatt et al. (2021a)	SOLOIST-WHF	1,222	T2D, HF	68.6 ± 9.5	69.3 ± 8.8	32.6	34.9	Sotagliflozin from 200 mg to 400 mg	21.9 months
NCT03315143	Bhatt et al. (2021b)	SCORED	10,584	T2D, CKD	68.4 ± 8.4	68.2 ± 8.4	44.3	45.5	Sotagliflozin from 200 mg to 400 mg	30 months
NCT03066830	Clinical trail (2021a)	——	507	T2D	63.3 ± 8.8	63.0 ± 9.9	41.1	48.8	Sotagliflozin 400 mg	79 weeks
NCT02926950	Clinical trail (2021b)	——	518	T2D	60.0 ± 10.1	59.9 ± 9.4	45.2	43.6	Sotagliflozin 400 mg	83 weeks
NCT03285594	Clinical trail (2021c)	SOTA-INS	571	T2D	62.5 ± 9.5	62.2 ± 8.9	46.1	40.3	Sotagliflozin 200mg and 400 mg	57.5 weeks
NCT03332771	Clinical trail (2021d)	SOTA-GLIM	954	T2D	59.3 ± 10.2	58.8 ± 11.2	48.6	48.4	Sotagliflozin 200 mg and 400 mg	54 weeks
NCT02384941	Buse et al. (2018)	inTandem1	793	T1D	46.5 ± 13.3	45.2 ± 12.7	53.1	48.9	Sotagliflozin 200 mg and 400 mg	52 weeks
NCT04065841	Clinical trail (2024)	ELIVATE	234	NASH and Liver Fibrosis	56.0 ± 12.1	54.9 ± 10.2	61.8	63.4	Licogliflozin 30 mg	52 weeks

AMI, acute myocardial infarction; CKD, chronic kidney disease; T1D, type 1 diabetes mellitus; T2D, type 2 diabetes mellitus; HF, heart failure; HFrEF, heart failure with reduced ejection fraction; HFpEF, heart failure with preserved ejection fraction; NASH, nonalcoholic steatohepatitis.

### 3.3 Meta-analysis

#### 3.3.1 The effect of SGLT2 inhibitors on AF/AFL

**FIGURE 3:**
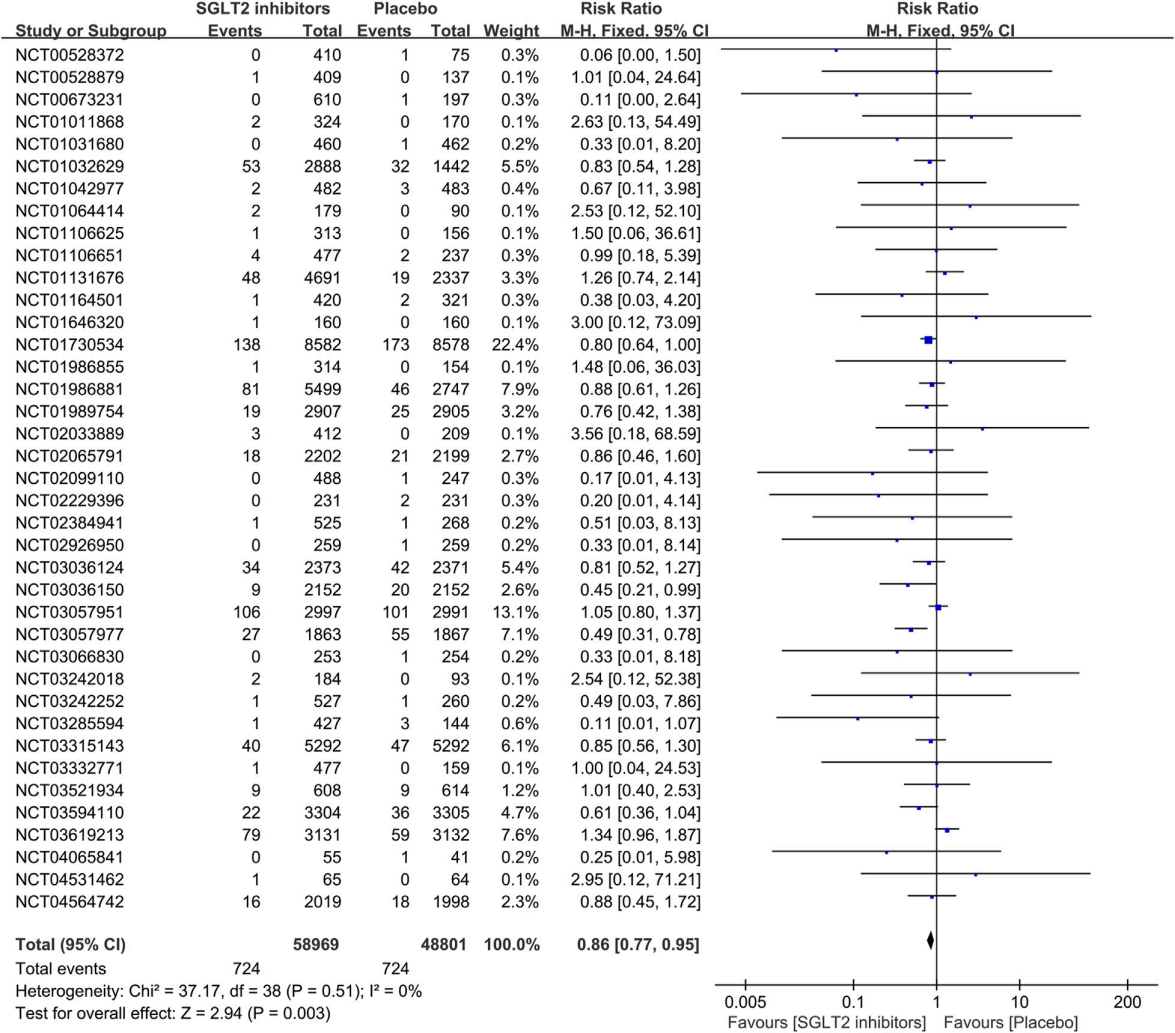
Forest plot comparing AF/AFL occurrence between SGLT2 inhibitors group and placebo group. SGLT2, sodium-glucose co-transporter 2; M-H, Mantel–Haenszel test; fixed, fixed-effects model; CI, confidence interval.

**FIGURE 4:**
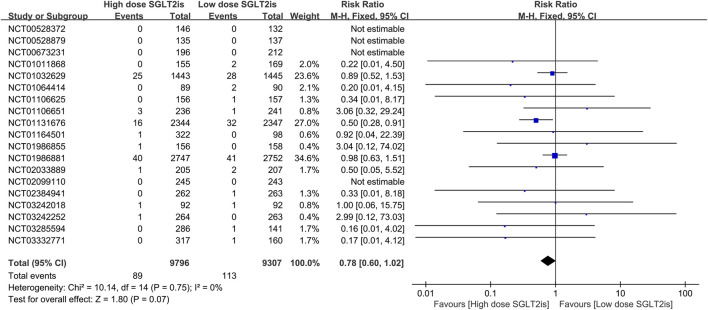
Forest plot comparing AF/AFL occurrence between high dose SGLT2 inhibitors and low dose SGLT2 inhibitors. SGLT2, sodium-glucose co-transporter 2; M-H, Mantel–Haenszel test; fixed, fixed-effects model; CI, confidence interval.

All 39 included RCTs reported on AF/AFL events. The meta-analysis revealed that patients treated with SGLT2 inhibitors had a reduced risk of AF/AFL compared with placebo (RR 0.86; 95%CI, 0.77–0.95; I^2^ = 0%; P = 0.003) (Figure 3). In 19 RCTS, SGLT2 inhibitors were grouped by dosage (dapagliflozin 10 mg/5 mg, empagliflozin 25 mg/10 mg, canagliflozin 300 mg/100 mg, ertugliflozin 15 mg/5 mg, sotagliflozin 400 mg/200 mg). Meta-analysis of high-dose versus low-dose SGLT2 inhibitors showed no statistically significant difference in the risk of AF/AFL (RR 0.78; 95%CI, 0.60–1.02; I^2^ = 0%; P = 0.07), although a decreasing trend was observed in the high-dose group (Figure 4). Of all trials, 31 enrolled patients with DM (30 with Type2 DM, 1 with Type 1 DM), 5 with HF (2 with HFrEF, 2 with HFpEF and 1 with HF of unspecified classification), and 8 with CKD (8 with both DM and CKD and 1 with CKD only). The RCTs included in this study involved different participant populations, with 31 focusing on DM, 5 on HF, and 8 on CKD. We conducted separate meta-analyses for each population subgroup. The result revealed that SGLT2 inhibitors significantly reduced AF/AFL risk compared to placebo in both DM (RR 0.84; 95%CI, 0.73–0.96; I^2^ = 0%; P = 0.01) and CKD patients (RR 0.72; 95%CI, 0.55–0.94; I^2^ = 0%; P = 0.02), but showed no significant effect in HF patients (RR 0.90; 95%CI, 0.64–1.27; I^2^ = 69%; P = 0.56) (Supplementary Figures S1–S3).

#### 3.3.2 The effect of SGLT2 inhibitors on VT, VF, sinus bradycardia

**FIGURE 5:**
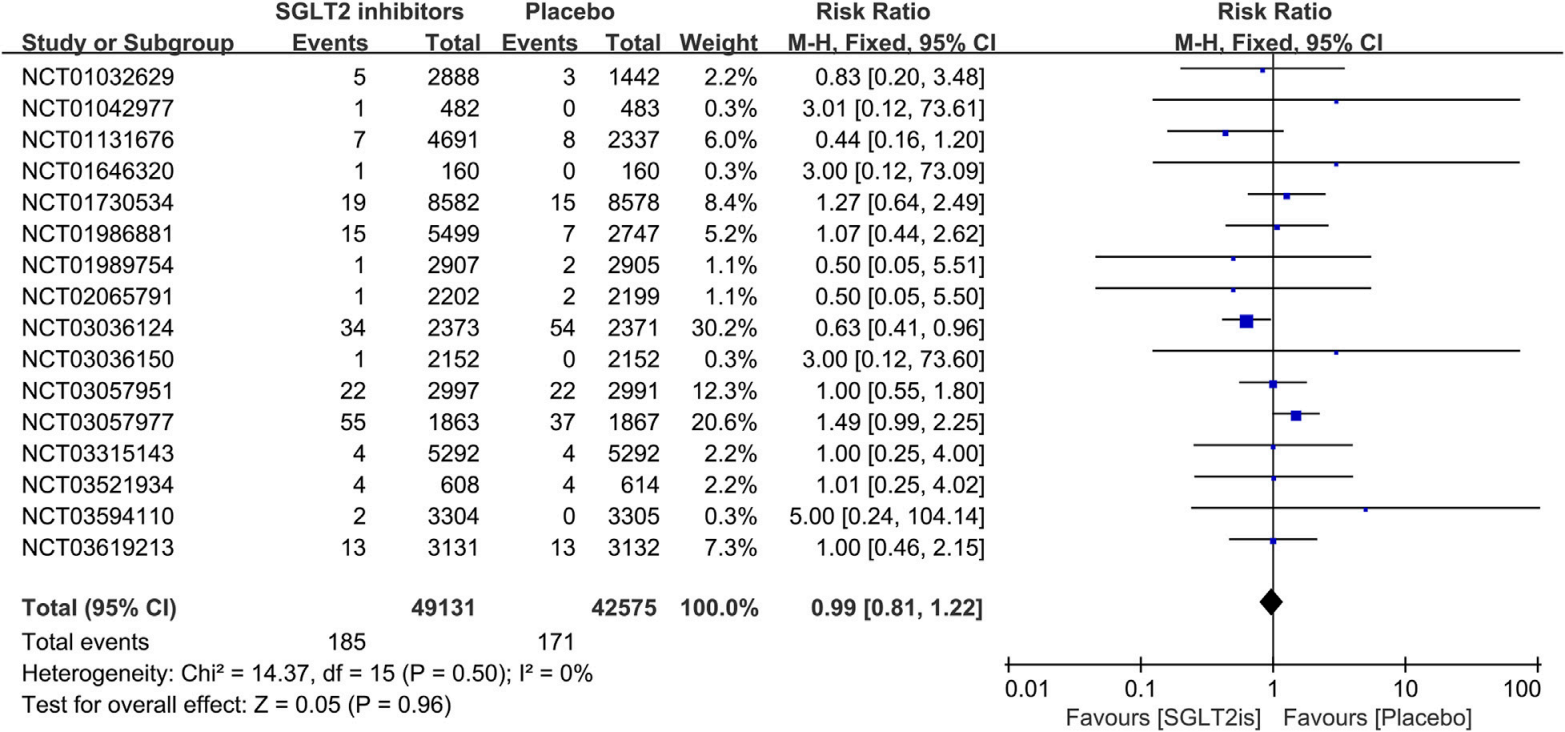
Forest plot comparing VT occurrence between SGLT2 inhibitors group and placebo group. SGLT2, sodium-glucose co-transporter 2; M-H, Mantel–Haenszel test; fixed, fixed-effects model; CI, confidence interval.

**FIGURE 6:**
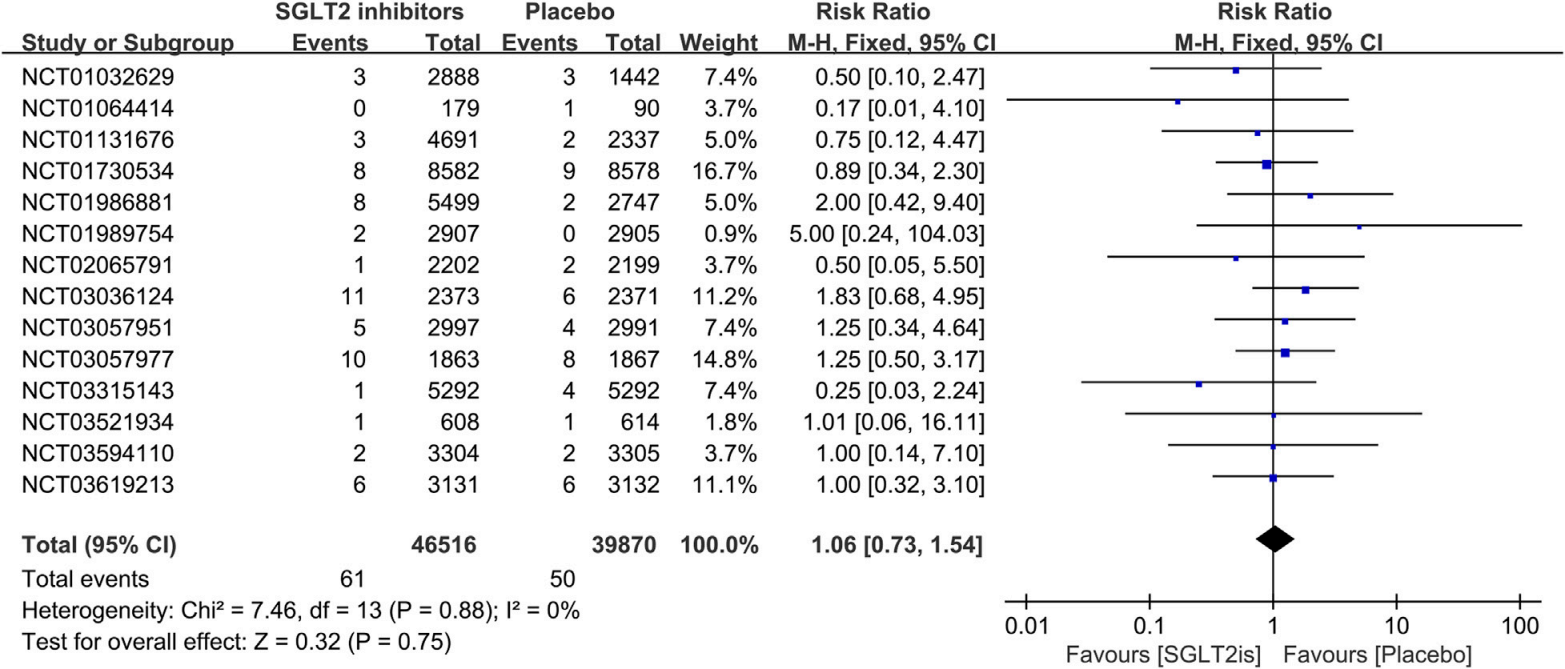
Forest plot comparing VF occurrence between SGLT2 inhibitors group and placebo group. SGLT2, sodium-glucose co-transporter 2; M-H, Mantel–Haenszel test; fixed, fixed-effects model; CI, confidence interval.

**FIGURE 7:**
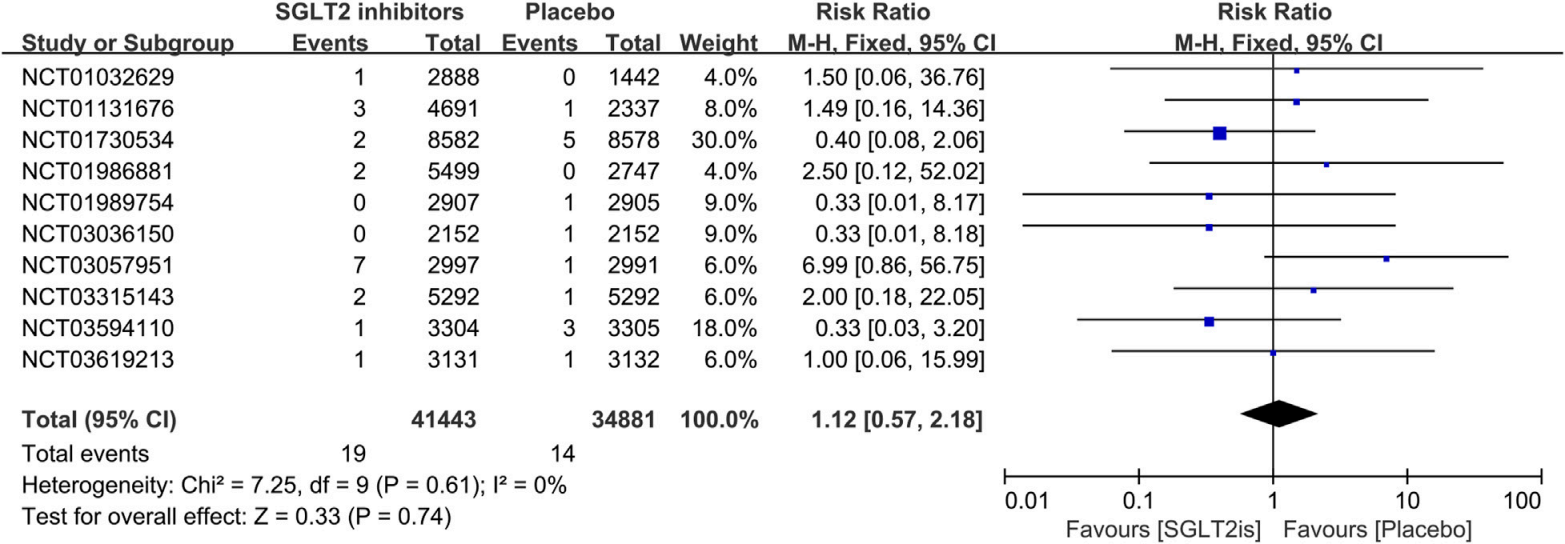
Forest plot comparing sinus bradycardia occurrence between SGLT2 inhibitors group and placebo group. SGLT2, sodium-glucose co-transporter 2; M-H, Mantel–Haenszel test; fixed, fixed-effects model; CI, confidence interval.

16 RCTs reported on VT events. The meta-analysis showed there was no significant difference in the risk of VT between the SGLT2 inhibitors group and the placebo group (RR 0.99; 95%CI, 0.81–1.22; I^2^ = 0%; P = 0.96) (Figure 5). Similarly, 14 RCTs reported on VF events, with no significant difference observed (RR 1.06; 95%CI, 0.73–1.54; I^2^ = 0%; P = 0.75) (Figure 6). 8 RCTs reported on sinus bradycardia events, and again, no significant difference was identified (RR 1.12; 95%CI, 0.57–2.18; I^2^ = 0%; P = 0.74) (Figure 7).

### 3.4 Sensitivity analysis and publication bias

Sensitivity analysis was conducted using the leave-one-out method. We found that the results of meta-analysis of AF/AFL, VT, VF, and sinus bradycardia were robust and not influenced by any single study. However, in the model evaluating the effect of different doses of SGLT2 inhibitors on AF/AFL, a statistically significant result was observed after excluding NCT01986881 (RR 0.68; 95%CI, 0.48–0.96; I^2^ = 0%; P = 0.03).

**FIGURE 8:**
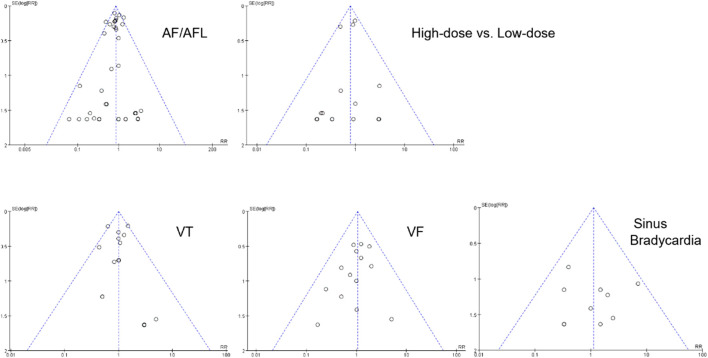
Funnel plot.

The funnel plots were symmetrical, suggested that the probability of publication bias is low (Figure 8).

## 4 Discussion

Atrial fibrillation, the most common arrhythmia worldwide with an increasing incidence (Joglar et al., 2024), is strongly associated with diabetes mellitus. The diabetic state facilitates the maintenance of AF by inducing atrial structural and electrical remodeling (Karam et al., 2017). SGLT2 inhibitors, a novel class of hypoglycemic agents, present a potential avenue for mitigating AF/AFL. A *post hoc* analysis derived from the large randomized controlled clinical trial DECLARE-TIMI58 reported that dapagliflozin reduced AF/AFL adverse events in patients with type 2 diabetes irrespective of a history of AF/AFL, atherosclerotic cardiovascular disease or HF (Zelniker et al., 2020). Metabolic remodeling is a catalyst for the initiation and perpetuation of AF (Bode et al., 2024). 2024 ESC Guidelines for the management of atrial fibrillation recommend effective glycemic control is beneficial for reducing burden, recurrence, and progression of AF in individuals with diabetes mellitus and AF (class IC) (Van Gelder et al., 2024). However, the evidence supporting this recommendation remains relatively limited.

Our meta-analysis of 39 RCTs demonstrated that patients treated with SGLT2 inhibitors had a significantly lower risk of AF/AFL compared with those receiving placebo (RR 0.86; 95%CI, 0.77–0.95; P = 0.003). This finding aligns with some previous meta-analyses (Li et al., 2021; Pandey et al., 2021). Differently, to assess the long-term effect of SGLT2 inhibitors on the risk of AF/AFL more comprehensively and accurately, we included only RCTs with a follow-up duration of at least 52 weeks, along with some unpublished raw data from ClinicalTrials.gov (NCT03066830, NCT02926950, NCT03285594, NCT03332771, and NCT04065841), and incorporated the most recent publications. Furthermore, our study directly compared the effects of high-dose and low-dose SGLT2 inhibitors on AF/AFL risk. While a decreasing trend was noted in the high-dose group, the difference was not statistically significant. Interestingly, sensitivity analysis revealed that the high-dose group had a statistically significant reduction in AF/AFL risk after excluding NCT01986881 (RR 0.68; 95%CI, 0.48–0.96; P = 0.03). This sensitivity analysis result should be interpreted cautiously, as excluding a high-quality trial may introduce bias. An animal study showed that high-dose empagliflozin significantly reduced AF inducibility in diabetic rats, compared with low-dose empagliflozin (Shao et al., 2019), contrasting our findings. Notably, there was also a study that reported findings different from the conclusions of our research. Zhang et al. (2024) reported in a meta-analysis that SGLT2 inhibitors did not reduce the risk of AF occurrence, irrespective of follow-up duration, drug type or dose, or the patient population. While their meta-analysis included trials published up to July 2023, our study incorporated additional evidence from newly published trials in 2023–2024 as well as five unpublished trials. These expanded data sources might contribute to the observed differences in outcomes. So more relevant clinical trials are necessary to clarify whether dosing influences the efficacy of SGLT2 inhibitors against AF/AFL in the future. In addition to AF/AFL, our research explored the effects of SGLT2 inhibitors on other arrhythmias. Unfortunately, SGLT2 inhibitors had no significant improvement in the risks of VT, VF, and sinus bradycardia (P > 0.05).

The mechanisms underlying the beneficial effects of SGLT2 inhibitors on AF are still under investigation. Shao et al. (2019) demonstrated that empagliflozin could inhibit oxidative stress, improve mitochondrial function, alleviate left atrial fibrosis, and reduce the incidence of AF in diabetic rats. Notably, the high-dose empagliflozin group showed a more significant reduction in left atrial fibrosis and AF incidence compared to the low-dose group. In our previous study, we found that empagliflozin improved calcium dysregulation caused by myocardial ischemia-reperfusion (Azam et al., 2021), a factor known to play a crucial role in the electrophysiological mechanisms underlying AF. Endoplasmic reticulum (ER) stress-mediated apoptosis was a contributing factor to the development of AF (Shi et al., 2015). Dapagliflozin has been shown to significantly suppress ER stress and cardiac fibrosis, reduce the incidence of AF and shorten the duration of AF in mitral regurgitation rats (Lin et al., 2021). Epicardial adipose tissue (EAT), a unique fat reservoir located between the myocardium and the epicardial visceral layer, is both a risk factor and an independent predictor for the occurrence and recurrence of AF after ablation. Mechanisms contributing to the occurrence of AF include genetic and neurological factors, inflammation, oxidative stress, fibrosis, fat infiltration, and atrial electrical or structural remodeling (Iacobellis, 2022; Wong et al., 2017). Empagliflozin has been shown to alleviate EAT inflammation by reducing GAPDH malonylation through downregulation of ACC1 expression, thereby attenuating atrial fibrosis (Li et al., 2023). Similarly, Badreldin et al. (2024) found that empagliflozin could protect the heart from AF in rats by inhibiting the NF-κB/HIF-1α regulatory axis and atrial remodeling. These animal studies provide further support for our conclusion that SGLT2 inhibitors have a beneficial impact on AF.

SGLT2 inhibitors, as part of guideline-directed medical therapy for patients with HFrEF, are now widely used in clinical practice, particularly dapagliflozin and empagliflozin. Data from the OpTIMa-HF registry, an observational, multicenter, real-world study of Italian HFrEF patients, demonstrate their rapid adoption: 63.2% of patients were prescribed SGLT2 inhibitors, with dapagliflozin (76%) and empagliflozin (23.6%) being the most common, while canagliflozin and ertugliflozin accounted for only 0.4%. These findings underscore the swift integration of SGLT2 inhibitors into routine HFrEF management (Paolillo et al., 2025). Some animal studies have shown their beneficial effects on atrial electrophysiology in heart failure models. Bode et al. (2021) found that sotagliflozin ameliorated left atrial (LA) remodeling in metabolic HFpEF. It also improved key features of Ca2+-mediated cellular arrhythmogenesis in LA cardiomyocytes, such as the magnitude of spontaneous Ca^2+^ release events (SCaEs), mitochondrial Ca^2+^ buffering capacity, diastolic calcium accumulation, and sodium-calcium exchanger (NCX) activity. Trum et al. (2024) isolated cardiomyocytes from atrial biopsies of HFpEF or non-HF patients undergoing elective cardiac surgery. They found increased Na influx in human atrial cardiomyocytes from HFpEF patients, partly due to an increase late sodium current (late I_Na_), and this increase was associated with AF susceptibility (Zhang et al., 2017). Notably, empagliflozin significantly reduced both Na + influx and late I_Na_ (Trum et al., 2024), suggesting a potential therapeutic benefit for AF in HFpEF. Despite these promising mechanistic insights, our meta-analysis failed to demonstrate significant AF/AFL risk reduction in HF patients, possibly due to the limited number of RCTs focusing specifically on HF cohorts and substantial heterogeneity observed among these trials (I^2^>50%). Therefore, further well-designed RCTs in HF subgroups are needed to validate these results and assess whether SGLT2 inhibitors reduce AF/AFL risk in this population.

This meta-analysis has some limitations that warrant consideration. Firstly, in the vast majority of the included RCTs, arrhythmia events were reported as adverse events rather than primary or secondary outcomes. Secondly, some RCTs lacked detailed reporting on the arrhythmia history of participants, such as paroxysmal AF/AFL and persistent AF/AFL, which might complicate the accurate evaluation of SGLT2 inhibitors’ effects.

In summary, our meta-analysis demonstrates that SGLT2 inhibitors significantly reduce the risk of AF/AFL but have no notable impact on the risk of VT, VF, and sinus bradycardia. Furthermore, our study found no statistically significant dose-dependent differences in AF/AFL incidence. In the future, large-scale randomized controlled clinical trials focusing on SGLT2 inhibitors and arrhythmias are needed to validate our findings.

## Data Availability

The original contributions presented in the study are included in the article/Supplementary Material, further inquiries can be directed to the corresponding authors.

## References

[B1] Al-Sharea A. Murphy A. J. Huggins L. A. Goldberg I. J. Nagareddy P. R. (2018). SGLT2 inhibition reduces atherosclerosis by enhancing lipoprotein clearance in Ldlr(-/-) type 1 diabetic mice. Atherosclerosis 271, 166–176. 10.1016/j.atherosclerosis.2018.02.028 29518749 PMC7196281

[B2] Anker S. D. Butler J. Filippatos G. Ferreira J. P. Bocchi E. Böhm M. (2021). Empagliflozin in heart failure with a preserved ejection fraction. N. Engl. J. Med. 385 (16), 1451–1461. 10.1056/NEJMoa2107038 34449189

[B3] Azam M. A. Chakraborty P. Si D. Du B. Massé S. Lai P. F. H. (2021). Anti-arrhythmic and inotropic effects of empagliflozin following myocardial ischemia. Life Sci. 276, 119440. 10.1016/j.lfs.2021.119440 33781832

[B4] Badreldin H. Elshal M. El-Karef A. Ibrahim T. (2024). Empagliflozin protects the heart from atrial fibrillation in rats through inhibiting the NF-κB/HIF-1α regulatory axis and atrial remodeling. Int. Immunopharmacol. 143 (Pt 2), 113403. 10.1016/j.intimp.2024.113403 39437485

[B5] Bailey C. J. Gross J. L. Hennicken D. Iqbal N. Mansfield T. A. List J. F. (2013). Dapagliflozin add-on to metformin in type 2 diabetes inadequately controlled with metformin: a randomized, double-blind, placebo-controlled 102-week trial. BMC Med. 11, 43. 10.1186/1741-7015-11-43 23425012 PMC3606470

[B6] Bailey C. J. Morales Villegas E. C. Woo V. Tang W. Ptaszynska A. List J. F. (2015). Efficacy and safety of dapagliflozin monotherapy in people with type 2 diabetes: a randomized double-blind placebo-controlled 102-week trial. Diabet. Med. 32 (4), 531–541. 10.1111/dme.12624 25381876

[B7] Barnett A. H. Mithal A. Manassie J. Jones R. Rattunde H. Woerle H. J. (2014). Efficacy and safety of empagliflozin added to existing antidiabetes treatment in patients with type 2 diabetes and chronic kidney disease: a randomised, double-blind, placebo-controlled trial. Lancet Diabetes Endocrinol. 2 (5), 369–384. 10.1016/S2213-8587(13)70208-0 24795251

[B8] Bhatt D. L. Szarek M. Pitt B. Cannon C. P. Leiter L. A. McGuire D. K. (2021a). Sotagliflozin in patients with diabetes and chronic kidney disease. N. Engl. J. Med. 384 (2), 129–139. 10.1056/NEJMoa2030186 33200891

[B9] Bhatt D. L. Szarek M. Steg P. G. Cannon C. P. Leiter L. A. McGuire D. K. (2021b). Sotagliflozin in patients with diabetes and recent worsening heart failure. N. Engl. J. Med. 384 (2), 117–128. 10.1056/NEJMoa2030183 33200892

[B10] Bode B. Stenlöf K. Sullivan D. Fung A. Usiskin K. (2013). Efficacy and safety of canagliflozin treatment in older subjects with type 2 diabetes mellitus: a randomized trial. Hosp. Pract. 41 (2), 72–84. 10.3810/hp.2013.04.1020 23680739

[B11] Bode D. Pronto J. R. D. Schiattarella G. G. Voigt N. (2024). Metabolic remodelling in atrial fibrillation: manifestations, mechanisms and clinical implications. Nat. Rev. Cardiol. 21 (10), 682–700. 10.1038/s41569-024-01038-6 38816507

[B12] Bode D. Semmler L. Wakula P. Hegemann N. Primessnig U. Beindorff N. (2021). Dual SGLT-1 and SGLT-2 inhibition improves left atrial dysfunction in HFpEF. Cardiovasc Diabetol. 20 (1), 7. 10.1186/s12933-020-01208-z 33413413 PMC7792219

[B13] Buse J. B. Garg S. K. Rosenstock J. Bailey T. S. Banks P. Bode B. W. (2018). Sotagliflozin in combination with optimized insulin therapy in adults with type 1 diabetes: the north American inTandem1 study. Diabetes Care 41 (9), 1970–1980. 10.2337/dc18-0343 29937430 PMC6105319

[B14] Cannon C. P. Pratley R. Dagogo-Jack S. Mancuso J. Huyck S. Masiukiewicz U. (2020). Cardiovascular outcomes with ertugliflozin in type 2 diabetes. N. Engl. J. Med. 383 (15), 1425–1435. 10.1056/NEJMoa2004967 32966714

[B15] Cefalu W. T. Leiter L. A. De Bruin T. W. Gause-Nilsson I. Sugg J. Parikh S. J. (2015). Dapagliflozin's effects on glycemia and cardiovascular risk factors in high-risk patients with type 2 diabetes: a 24-Week, multicenter, randomized, double-blind, placebo-controlled study with a 28-Week extension. Diabetes Care 38 (7), 1218–1227. 10.2337/dc14-0315 25852208 PMC4831907

[B16] Cherney D. Z. I. Ferrannini E. Umpierrez G. E. Peters A. L. Rosenstock J. Carroll A. K. (2021). Efficacy and safety of sotagliflozin in patients with type 2 diabetes and severe renal impairment. Diabetes Obes. Metab. 23 (12), 2632–2642. 10.1111/dom.14513 34338408

[B17] Cherney D. Z. I. Ferrannini E. Umpierrez G. E. Peters A. L. Rosenstock J. Powell D. R. (2023). Efficacy and safety of sotagliflozin in patients with type 2 diabetes and stage 3 chronic kidney disease. Diabetes Obes. Metab. 25 (6), 1646–1657. 10.1111/dom.15019 36782093

[B18] Clinical trail (2021a). Efficacy and safety of sotagliflozin versus glimepiride and placebo in participants with type 2 diabetes mellitus that are taking metformin monotherapy (SOTA-GLIM). Available online at: https://clinicaltrials.gov/study/NCT03332771?term=NCT&rank=1.

[B19] Clinical trail (2021b). Efficacy and safety of sotagliflozin versus placebo in participants with type 2 diabetes mellitus on background of sulfonylurea alone or with metformin. Available online at: https://clinicaltrials.gov/study/NCT03066830?term=NCT&rank=1.

[B20] Clinical trail (2021c). Efficacy and safety of sotagliflozin versus placebo in participants with type 2 diabetes mellitus who have in adequate glycemic control while taking insulin alone or with other oral antidiabetic agents (SOTA-INS). Available online at: https://clinicaltrials.gov/study/NCT03285594?term=NCT&rank=1.

[B21] Clinical trail (2021d). Efficacy and safety of sotagliflozin versus placebo in patients with type 2 diabetes mellitus on background of metformin. Available online at: https://clinicaltrials.gov/study/NCT02926950?term=NCT&rank=1.

[B22] Clinical trail (2024). Efficacy safety and tolerability of the combination of tropifexor and licogliflozin and each monotherapy, compared with placebo in adult patients with NASH and Liver Fibrosis (ELIVATE). Available online at: https://clinicaltrials.gov/study/NCT04065841?term=NCT&rank=1.

[B23] Ganbaatar B. Fukuda D. Shinohara M. Yagi S. Kusunose K. Yamada H. (2020). Empagliflozin ameliorates endothelial dysfunction and suppresses atherogenesis in diabetic apolipoprotein E-deficient mice. Eur. J. Pharmacol. 875, 173040. 10.1016/j.ejphar.2020.173040 32114052

[B24] Grunberger G. Camp S. Johnson J. Huyck S. Terra S. G. Mancuso J. P. (2018). Ertugliflozin in patients with stage 3 chronic kidney disease and type 2 diabetes mellitus: the VERTIS RENAL randomized study. Diabetes Ther. 9 (1), 49–66. 10.1007/s13300-017-0337-5 29159457 PMC5801223

[B25] Heerspink H. J. L. StefáNSSON B. V. Correa-Rotter R. Chertow G. M. Greene T. Hou F. F. (2020). Dapagliflozin in patients with chronic kidney disease. N. Engl. J. Med. 383 (15), 1436–1446. 10.1056/NEJMoa2024816 32970396

[B26] Heidenreich P. A. Bozkurt B. Aguilar D. Allen L. A. Byun J. J. Colvin M. M. (2022). 2022 AHA/ACC/HFSA guideline for the management of heart failure: a report of the American college of cardiology/american heart association joint committee on clinical practice guidelines. Circulation 145 (18), e895–e1032. 10.1161/CIR.0000000000001063 35363499

[B27] Herrington W. G. Staplin N. Wanner C. Green J. B. Hauske S. J. Emberson J. R. (2023). Empagliflozin in patients with chronic kidney disease. N. Engl. J. Med. 388 (2), 117–127. 10.1056/NEJMoa2204233 36331190 PMC7614055

[B28] Iacobellis G. (2022). Epicardial adipose tissue in contemporary cardiology. Nat. Rev. Cardiol. 19 (9), 593–606. 10.1038/s41569-022-00679-9 35296869 PMC8926097

[B29] Jabbour S. A. FríAS J. P. Ahmed A. Hardy E. Choi J. Sjöström C. D. (2020). Efficacy and safety over 2 years of exenatide plus dapagliflozin in the DURATION-8 study: a multicenter, double-blind, phase 3, randomized controlled trial. Diabetes Care 43 (10), 2528–2536. 10.2337/dc19-1350 32816874 PMC7510043

[B30] James S. Erlinge D. Storey R. F. McGuire D. K. de Belder M. Eriksson N. (2024). Dapagliflozin in myocardial infarction without diabetes or heart failure. NEJM Evid. 3 (2), EVIDoa2300286. 10.1056/EVIDoa2300286 38320489

[B31] Joglar J. A. Chung M. K. Armbruster A. L. Benjamin E. J. Chyou J. Y. Edmond M. C. (2024). 2023 ACC/AHA/ACCP/HRS guideline for the diagnosis and management of atrial fibrillation: a report of the American college of cardiology/american heart association joint committee on clinical practice guidelines. J. Am. Coll. Cardiol. 83 (1), 109–279. 10.1016/j.jacc.2023.08.017 38043043 PMC11104284

[B32] Karam B. S. Chavez-Moreno A. Koh W. Akar J. G. Akar F. G. (2017). Oxidative stress and inflammation as central mediators of atrial fibrillation in obesity and diabetes. Cardiovasc Diabetol. 16 (1), 120. 10.1186/s12933-017-0604-9 28962617 PMC5622555

[B33] Leiter L. A. Cefalu W. T. De Bruin T. W. Gause-Nilsson I. Sugg J. Parikh S. J. (2014). Dapagliflozin added to usual care in individuals with type 2 diabetes mellitus with preexisting cardiovascular disease: a 24-week, multicenter, randomized, double-blind, placebo-controlled study with a 28-week extension. J. Am. Geriatr. Soc. 62 (7), 1252–1262. 10.1111/jgs.12881 24890683

[B34] Li D. Liu Y. Hidru T. H. Yang X. Wang Y. Chen C. (2021). Protective effects of sodium-glucose transporter 2 inhibitors on atrial fibrillation and atrial flutter: a systematic review and Meta- analysis of randomized placebo-controlled trials. Front. Endocrinol. (Lausanne) 12, 619586. 10.3389/fendo.2021.619586 33815278 PMC8018283

[B35] Li L. Hua C. Liu X. Wang Y. Zhao L. Zhang Y. (2023). SGLT2i alleviates epicardial adipose tissue inflammation by modulating ketone body-glyceraldehyde-3-phosphate dehydrogenase malonylation pathway. J. Cardiovasc Med. Hagerst. 24 (4), 232–243. 10.2459/JCM.0000000000001453 36938811

[B36] Lin Y. W. Chen C. Y. Shih J. Y. Cheng B. C. Chang C. P. Lin M. T. (2021). Dapagliflozin improves cardiac hemodynamics and mitigates arrhythmogenesis in mitral regurgitation-induced myocardial dysfunction. J. Am. Heart Assoc. 10 (7), e019274. 10.1161/JAHA.120.019274 33749310 PMC8174384

[B37] Mathieu C. Ranetti A. E. Li D. Ekholm E. Cook W. Hirshberg B. (2015). Randomized, double-blind, phase 3 trial of triple therapy with dapagliflozin Add-on to saxagliptin plus metformin in type 2 diabetes. Diabetes Care 38 (11), 2009–2017. 10.2337/dc15-0779 26246458

[B38] Mc Causland F. R. Claggett B. L. Vaduganathan M. Desai A. S. Jhund P. de Boer R. A. (2023). Dapagliflozin and kidney outcomes in patients with heart failure with mildly reduced or preserved ejection fraction: a prespecified analysis of the DELIVER randomized clinical trial. JAMA Cardiol. 8 (1), 56–65. 10.1001/jamacardio.2022.4210 36326604 PMC9634592

[B39] Mcdonagh T. A. Metra M. Adamo M. Gardner R. S. Baumbach A. Bohm H. (2022). 2021 ESC guidelines for the diagnosis and treatment of acute and chronic heart failure: developed by the task force for the diagnosis and treatment of acute and chronic heart failure of the european society of cardiology (ESC). With the special contribution of the heart failure association (HFA) of the ESC. Eur. J. Heart Fail 24 (1), 4–131. 10.1002/ejhf.2333 35083827

[B40] Mcmurray J. J. V. Solomon S. D. Inzucchi S. E. Køber L. Kosiborod M. N. Martinez F. A. (2019). Dapagliflozin in patients with heart failure and reduced ejection fraction. N. Engl. J. Med. 381 (21), 1995–2008. 10.1056/NEJMoa1911303 31535829

[B41] Nakatsu Y. Kokubo H. Bumdelger B. Yoshizumi M. Yamamotoya T. Matsunaga Y. (2017). The SGLT2 inhibitor luseogliflozin rapidly normalizes aortic mRNA levels of inflammation-related but not lipid-metabolism-related genes and suppresses atherosclerosis in diabetic ApoE KO mice. Int. J. Mol. Sci. 18 (8), 1704. 10.3390/ijms18081704 28777298 PMC5578094

[B42] Nasiri-Ansari Ν. Dimitriadis G. K. Agrogiannis G. Perrea D. Kostakis I. D. Kaltsas G. (2018). Canagliflozin attenuates the progression of atherosclerosis and inflammation process in APOE knockout mice. Cardiovasc Diabetol. 17 (1), 106. 10.1186/s12933-018-0749-1 30049285 PMC6063004

[B43] Neal B. Perkovic V. Mahaffey K. W. de Zeeuw D. Fulcher G. Erondu N. (2017). Canagliflozin and cardiovascular and renal events in type 2 diabetes. N. Engl. J. Med. 377 (7), 644–657. 10.1056/NEJMoa1611925 28605608

[B44] Packer M. Anker S. D. Butler J. Filippatos G. Pocock S. J. Carson P. (2020). Cardiovascular and renal outcomes with empagliflozin in heart failure. N. Engl. J. Med. 383 (15), 1413–1424. 10.1056/NEJMoa2022190 32865377

[B45] Page M. J. Mckenzie J. E. Bossuyt P. M. Boutron I. Hoffmann T. C. Mulrow C. D. (2021). The PRISMA 2020 statement: an updated guideline for reporting systematic reviews. Bmj 372, n71. 10.1136/bmj.n71 33782057 PMC8005924

[B46] Pandey A. K. Okaj I. Kaur H. Belley-Cote E. P. Wang J. Oraii A. (2021). Sodium-glucose Co-Transporter inhibitors and atrial fibrillation: a systematic review and meta-analysis of randomized controlled trials. J. Am. Heart Assoc. 10 (17), e022222. 10.1161/JAHA.121.022222 34459238 PMC8649253

[B47] Paolillo S. Basile C. Marzano F. Bruzzese D. Agostoni P. Mattavelli I. (2025). Implementation of guideline-directed medical therapy in patients with heart failure with reduced ejection fraction (OpTIMa-HF registry). Esc. Heart Fail 12 (3), 1786–1795. 10.1002/ehf2.15172 39909062 PMC12055350

[B48] Perkovic V. Jardine M. J. Neal B. Bompoint S. Heerspink H. J. L. Charytan D. M. (2019). Canagliflozin and renal outcomes in type 2 diabetes and nephropathy. N. Engl. J. Med. 380 (24), 2295–2306. 10.1056/NEJMoa1811744 30990260

[B49] Pratley R. E. Eldor R. Raji A. Golm G. Huyck S. B. Qiu Y. (2018). Ertugliflozin plus sitagliptin versus either individual agent over 52 weeks in patients with type 2 diabetes mellitus inadequately controlled with metformin: the VERTIS FACTORIAL randomized trial. Diabetes Obes. Metab. 20 (5), 1111–1120. 10.1111/dom.13194 29266675 PMC5947297

[B50] Rosenstock J. Frias J. PáLL D. Charbonnel B. Pascu R. Saur D. (2018). Effect of ertugliflozin on glucose control, body weight, blood pressure and bone density in type 2 diabetes mellitus inadequately controlled on metformin monotherapy (VERTIS MET). Diabetes Obes. Metab. 20 (3), 520–529. 10.1111/dom.13103 28857451

[B51] Rosenstock J. Jelaska A. Zeller C. Kim G. Broedl U. C. Woerle H. J. (2015). Impact of empagliflozin added on to basal insulin in type 2 diabetes inadequately controlled on basal insulin: a 78-week randomized, double-blind, placebo-controlled trial. Diabetes Obes. Metab. 17 (10), 936–948. 10.1111/dom.12503 26040302 PMC5034797

[B52] Shao Q. Meng L. Lee S. Tse G. Gong M. Zhang Z. (2019). Empagliflozin, a sodium glucose co-transporter-2 inhibitor, alleviates atrial remodeling and improves mitochondrial function in high-fat diet/streptozotocin-induced diabetic rats. Cardiovasc Diabetol. 18 (1), 165. 10.1186/s12933-019-0964-4 31779619 PMC6882319

[B53] Shi J. Jiang Q. Ding X. Xu W. Wang D. W. Chen M. (2015). The ER stress-mediated mitochondrial apoptotic pathway and MAPKs modulate tachypacing-induced apoptosis in HL-1 atrial myocytes. PLoS One 10 (2), e0117567. 10.1371/journal.pone.0117567 25689866 PMC4331367

[B54] Trum M. Riechel J. Schollmeier E. Lebek S. Hegner P. Reuthner K. (2024). Empagliflozin inhibits increased Na influx in atrial cardiomyocytes of patients with HFpEF. Cardiovasc Res. 120 (9), 999–1010. 10.1093/cvr/cvae095 38728438 PMC11288740

[B55] Van Gelder I. C. Rienstra M. Bunting K. V. Casado-Arroyo R. Caso V. Crijns H. J. G. M. (2024). 2024 ESC guidelines for the management of atrial fibrillation developed in collaboration with the european association for cardio-thoracic surgery (EACTS). Eur. Heart J. 45 (36), 3314–3414. 10.1093/eurheartj/ehae176 39210723

[B56] Wei J. J. Du J. L. (2023). Mechanisms of sodium-glucose cotransporter 2 inhibitors in heart failure. Cardiovasc. INNOVATIONS Appl. 8 (1). 10.15212/cvia.2023.0028

[B57] Wilding J. P. Charpentier G. Hollander P. González-Gálvez G. Mathieu C. Vercruysse F. (2013). Efficacy and safety of canagliflozin in patients with type 2 diabetes mellitus inadequately controlled with metformin and sulphonylurea: a randomised trial. Int. J. Clin. Pract. 67 (12), 1267–1282. 10.1111/ijcp.12322 24118688 PMC4282288

[B58] Wilding J. P. Woo V. Rohwedder K. Sugg J. Parikh S. Dapagliflozin 006 Study Group (2014). Dapagliflozin in patients with type 2 diabetes receiving high doses of insulin: efficacy and safety over 2 years. Diabetes Obes. Metab. 16 (2), 124–136. 10.1111/dom.12187 23911013

[B59] Wiviott S. D. Raz I. Bonaca M. P. Mosenzon O. Kato E. T. Cahn A. (2019). Dapagliflozin and cardiovascular outcomes in type 2 diabetes. N. Engl. J. Med. 380 (4), 347–357. 10.1056/NEJMoa1812389 30415602

[B60] Wong C. X. Ganesan A. N. Selvanayagam J. B. (2017). Epicardial fat and atrial fibrillation: current evidence, potential mechanisms, clinical implications, and future directions. Eur. Heart J. 38 (17), 1294–1302. 10.1093/eurheartj/ehw045 26935271

[B61] Yabe D. Shiki K. Suzaki K. Meinicke T. Kotobuki Y. Nishida K. (2021). Rationale and design of the EMPA-ELDERLY trial: a randomised, double-blind, placebo-controlled, 52-week clinical trial of the efficacy and safety of the sodium-glucose cotransporter-2 inhibitor empagliflozin in elderly Japanese patients with type 2 diabetes. BMJ Open 11 (4), e045844. 10.1136/bmjopen-2020-045844 PMC8031078 33827843

[B62] Yale J. F. Bakris G. Cariou B. Nieto J. David-Neto E. Yue D. (2014). Efficacy and safety of canagliflozin over 52 weeks in patients with type 2 diabetes mellitus and chronic kidney disease. Diabetes Obes. Metab. 16 (10), 1016–1027. 10.1111/dom.12348 24965700

[B63] Zelniker T. A. Bonaca M. P. Furtado R. H. M. Mosenzon O. Kuder J. F. Murphy S. A. (2020). Effect of dapagliflozin on atrial fibrillation in patients with type 2 diabetes mellitus: insights from the DECLARE-TIMI 58 trial. Circulation 141 (15), 1227–1234. 10.1161/CIRCULATIONAHA.119.044183 31983236

[B64] Zhang H. D. Ding L. Mi L. J. Zhang A. K. Zhang K. Jiang Z. H. (2024). Sodium-glucose co-transporter-2 inhibitors for the prevention of atrial fibrillation: a systemic review and meta-analysis. Eur. J. Prev. Cardiol. 31 (7), 770–779. 10.1093/eurjpc/zwad356 37966828

[B65] Zhang Y. Wang H. M. Wang Y. Z. Jin X. X. Zhang Y. Y. Zhao Y. (2017). Increment of late sodium currents in the left atrial myocytes and its potential contribution to increased susceptibility of atrial fibrillation in castrated Male mice. Heart rhythm. 14 (7), 1073–1080. 10.1016/j.hrthm.2017.01.046 28185917

[B66] Zinman B. Wanner C. Lachin J. M. Fitchett D. Bluhmki E. Hantel S. (2015). Empagliflozin, cardiovascular outcomes, and mortality in type 2 diabetes. N. Engl. J. Med. 373 (22), 2117–2128. 10.1056/NEJMoa1504720 26378978

