# Supplementary material for: Long-term effects of SGLT2 inhibitors on arrhythmias: a systematic review and meta-analysis
Source: Front Pharmacol. 2025 Jul 2;16:1558367. doi: 10.3389/fphar.2025.1558367 (PMC12263634; doi:10.3389/fphar.2025.1558367)
Supplement: Supplementary file 1 [file DataSheet1.pdf]

Supplementary Materials

Supplemental Figure S1 Forest plot comparing AF/AFL occurrence between SGLT2 inhibitors and placebo in DM patients

Supplemental Figure S2 Forest plot comparing AF/AFL occurrence between SGLT2 inhibitors and placebo in CKD patients

Supplemental Figure S3 Forest plot comparing AF/AFL occurrence between SGLT2 inhibitors and placebo in HF patients

Supplemental Table S1 Comprehensive Search Strategy

Supplemental Figure S1 Forest plot comparing AF/AFL occurrence between SGLT2 inhibitors and placebo in DM patients

Study or Subgroup	SGLT2 inhibitors Events	Total	Placebo Events	Total	Weight	Risk Ratio M-H, Fixed, 95% CI
NCT00528372	0	410	1	75	0.6%	0.06 [0.00, 1.50]
NCT00528879	1	409	0	137	0.2%	1.01 [0.04, 24.64]
NCT00673231	0	610	1	197	0.5%	0.11 [0.00, 2.64]
NCT01011868	2	324	0	170	0.1%	2.63 [0.13, 54.49]
NCT01031680	0	460	1	462	0.3%	0.33 [0.01, 8.20]
NCT01032629	53	2888	32	1442	9.7%	0.83 [0.54, 1.28]
NCT01042977	2	482	3	483	0.7%	0.67 [0.11, 3.98]
NCT01064414	2	179	0	90	0.2%	2.53 [0.12, 52.10]
NCT01106625	1	313	0	156	0.2%	1.50 [0.06, 36.61]
NCT01106651	4	477	2	237	0.6%	0.99 [0.18, 5.39]
NCT01131676	48	4691	19	2337	5.8%	1.26 [0.74, 2.14]
NCT01164501	1	420	2	321	0.5%	0.38 [0.03, 4.20]
NCT01646320	1	160	0	160	0.1%	3.00 [0.12, 73.09]
NCT01730534	138	8582	173	8578	39.3%	0.80 [0.64, 1.00]
NCT01986855	1	314	0	154	0.2%	1.48 [0.06, 36.03]
NCT01986881	81	5499	46	2747	14.0%	0.88 [0.61, 1.26]
NCT01989754	19	2907	25	2905	5.7%	0.76 [0.42, 1.38]
NCT02033889	3	412	0	209	0.2%	3.56 [0.18, 68.59]
NCT02065791	18	2202	21	2199	4.8%	0.86 [0.46, 1.60]
NCT02099110	0	488	1	247	0.5%	0.17 [0.01, 4.13]
NCT02229396	0	231	2	231	0.6%	0.20 [0.01, 4.14]
NCT02384941	1	525	1	268	0.3%	0.51 [0.03, 8.13]
NCT02926950	0	259	1	259	0.3%	0.33 [0.01, 8.14]
NCT03066830	0	253	1	254	0.3%	0.33 [0.01, 8.18]
NCT03242018	2	184	0	93	0.2%	2.54 [0.12, 52.38]
NCT03242252	1	527	1	260	0.3%	0.49 [0.03, 7.86]
NCT03285594	1	427	3	144	1.0%	0.11 [0.01, 1.07]
NCT03315143	40	5292	47	5292	10.7%	0.85 [0.56, 1.30]
NCT03332771	1	477	0	159	0.2%	1.00 [0.04, 24.53]
NCT03521934	9	608	9	614	2.0%	1.01 [0.40, 2.53]
NCT04531462	1	65	0	64	0.1%	2.95 [0.12, 71.21]
**Total (95% CI)**		**41075**		**30944**	**100.0%**	**0.84 [0.73, 0.96]**
Total events	431		392			

Heterogeneity: Chi² = 17.54, df = 30 (P = 0.97); I² = 0%

Test for overall effect: Z = 2.51 (P = 0.01)

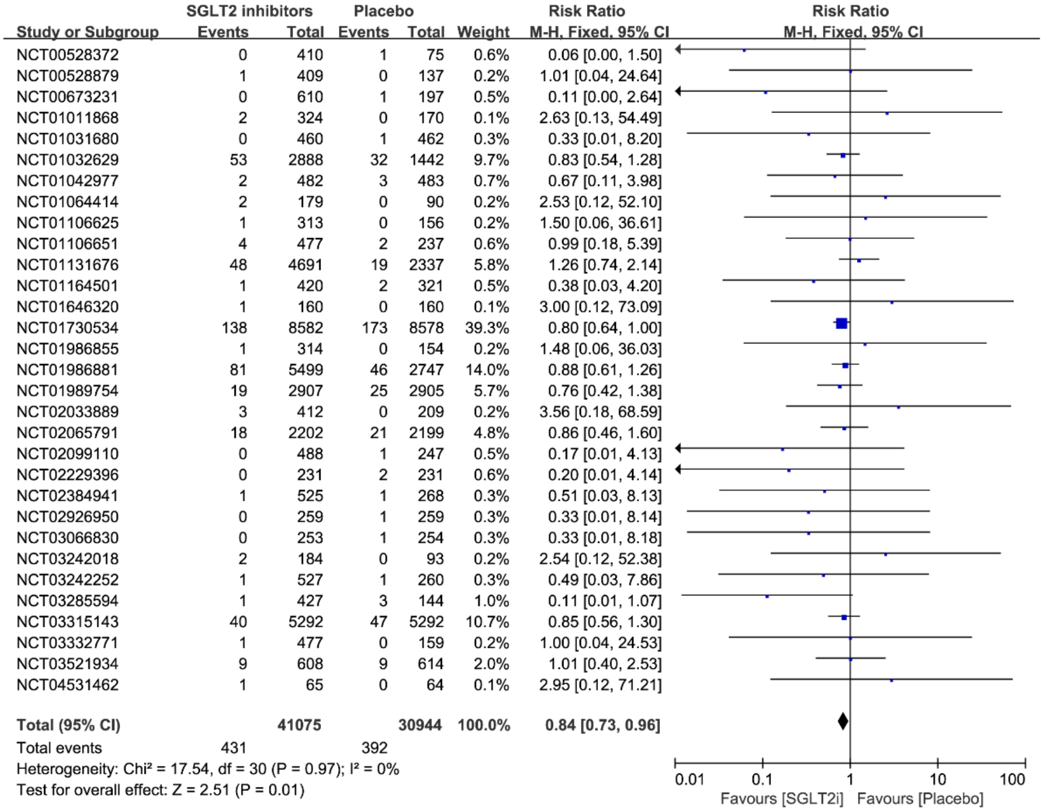

Supplemental Figure S2 Forest plot comparing AF/AFL occurrence between SGLT2 inhibitors and placebo in CKD patients

Study or Subgroup	SGLT2 inhibitors Events	SGLT2 inhibitors Total	Placebo Events	Placebo Total	Weight	Risk Ratio M-H, Fixed, 95% CI
NCT01164501	1	420	2	321	1.8%	0.38 [0.03, 4.20]
NCT01986855	1	314	0	154	0.5%	1.48 [0.06, 36.03]
NCT02065791	18	2202	21	2199	16.3%	0.86 [0.46, 1.60]
NCT03036150	9	2152	20	2152	15.5%	0.45 [0.21, 0.99]
NCT03242018	2	184	0	93	0.5%	2.54 [0.12, 52.38]
NCT03242252	1	527	1	260	1.0%	0.49 [0.03, 7.86]
NCT03315143	40	5292	47	5292	36.4%	0.85 [0.56, 1.30]
NCT03594110	22	3304	36	3305	27.9%	0.61 [0.36, 1.04]
**Total (95% CI)**		**14395**		**13776**	**100.0%**	**0.72 [0.55, 0.94]**
Total events	94		127			

Heterogeneity: Chi² = 3.85, df = 7 (P = 0.80); I² = 0%

Test for overall effect: Z = 2.40 (P = 0.02)

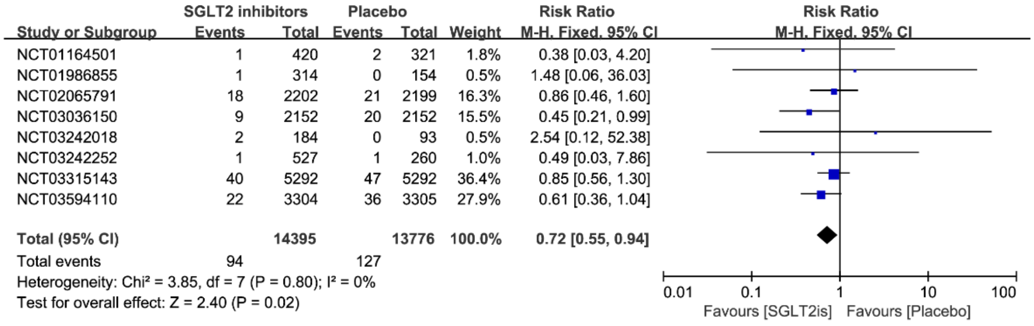

Supplemental Figure S3 Forest plot comparing AF/AFL occurrence between SGLT2 inhibitors and placebo in HF patients

Study or Subgroup	SGLT2 inhibitors Events	SGLT2 inhibitors Total	Placebo Events	Placebo Total	Weight	Risk Ratio M-H, Random, 95% CI
NCT03036124	34	2373	42	2371	20.2%	0.81 [0.52, 1.27]
NCT03057951	106	2997	101	2991	26.2%	1.05 [0.80, 1.37]
NCT03057977	27	1863	55	1867	20.0%	0.49 [0.31, 0.78]
NCT03521934	9	608	9	614	9.6%	1.01 [0.40, 2.53]
NCT03619213	79	3131	59	3132	24.0%	1.34 [0.96, 1.87]
**Total (95% CI)**		**10972**		**10975**	**100.0%**	**0.90 [0.64, 1.27]**
Total events	255		266			

Heterogeneity: Tau² = 0.10; Chi² = 13.06, df = 4 (P = 0.01); I² = 69%

Test for overall effect: Z = 0.58 (P = 0.56)

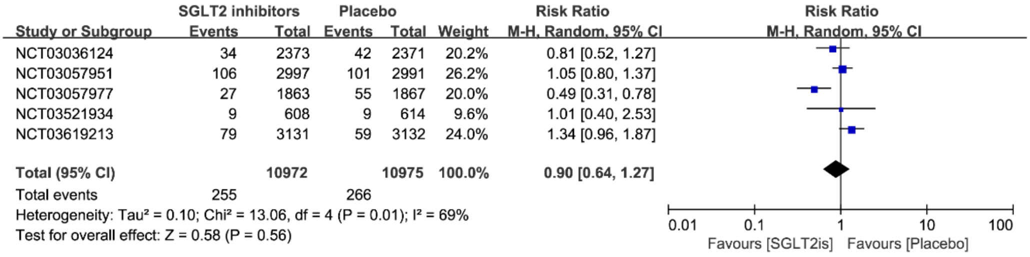

Supplemental Table S1 Comprehensive Search Strategy

Search Strategy of PubMed

(("Sodium-Glucose Transporter 2 Inhibitors"[MeSH Terms] OR ("Sodium-Glucose Transporter 2 Inhibitors"[Title/Abstract] OR "sodium glucose transporter 2 inhibitor"[Title/Abstract] OR "sodium glucose transporter 2 inhibitor"[Title/Abstract] OR "sglt 2 inhibitors"[Title/Abstract] OR "sglt 2 inhibitors"[Title/Abstract] OR "sglt2 inhibitors"[Title/Abstract] OR "sglt2 inhibitor"[Title/Abstract] OR "sglt 2 inhibitor"[Title/Abstract] OR "sglt 2 inhibitor"[Title/Abstract] OR "inhibitor sglt2"[Title/Abstract] OR "inhibitor sglt 2"[Title/Abstract] OR "Gliflozins"[Title/Abstract] OR "Gliflozin"[Title/Abstract]) OR ("Dapagliflozin"[Title/Abstract] OR "bms 512148"[Title/Abstract] OR "Empagliflozin"[Title/Abstract] OR "bi 10773"[Title/Abstract] OR "Canagliflozin"[Title/Abstract] OR "ta 7284"[Title/Abstract] OR "Tofogliflozin"[Title/Abstract] OR "Luseogliflozin"[Title/Abstract] OR "TS071"[Title/Abstract] OR "Ertugliflozin"[Title/Abstract] OR "PF04971729"[Title/Abstract] OR "Ipragliflozin"[Title/Abstract] OR "Remogliflozin"[Title/Abstract] OR "Sergliflozin"[Title/Abstract] OR "Sotagliflozin"[Title/Abstract] OR "LX4211"[Title/Abstract] OR "Licogliflozin"[Title/Abstract] OR "LIK066"[Title/Abstract])) AND ("atrial fibrillation"[MeSH Terms] OR ("atrial"[All Fields] AND "fibrillation"[All Fields]) OR "atrial fibrillation"[All Fields] OR ("atrial fibrillation"[MeSH Terms] OR ("atrial"[All Fields] AND "fibrillation"[All Fields]) OR "atrial fibrillation"[All Fields] OR ("atrial"[All Fields] AND "fibrillations"[All Fields]) OR "atrial fibrillations"[All Fields]) OR ("atrial fibrillation"[MeSH Terms] OR ("atrial"[All Fields] AND "fibrillation"[All Fields]) OR "atrial fibrillation"[All Fields] OR ("fibrillation"[All Fields] AND "atrial"[All Fields]) OR "fibrillation atrial"[All Fields]) OR ("atrial fibrillation"[MeSH Terms] OR ("atrial"[All Fields] AND "fibrillation"[All Fields]) OR "atrial fibrillation"[All Fields] OR ("fibrillations"[All Fields] AND "atrial"[All Fields]) OR "fibrillations atrial"[All Fields]) OR ("atrial fibrillation"[MeSH Terms] OR ("atrial"[All Fields] AND "fibrillation"[All Fields]) OR "atrial fibrillation"[All Fields] OR ("auricular"[All Fields] AND "fibrillation"[All Fields]) OR "auricular fibrillation"[All Fields]) OR ("atrial fibrillation"[MeSH Terms] OR ("atrial"[All Fields] AND "fibrillation"[All Fields]) OR "atrial fibrillation"[All Fields] OR ("auricular"[All Fields] AND "fibrillations"[All Fields]) OR "auricular fibrillations"[All Fields]) OR ("atrial fibrillation"[MeSH Terms] OR ("atrial"[All Fields] AND "fibrillation"[All Fields]) OR "atrial fibrillation"[All Fields] OR ("fibrillation"[All Fields] AND "auricular"[All Fields])) OR ("atrial fibrillation"[MeSH Terms] OR ("atrial"[All Fields] AND "fibrillation"[All Fields]) OR "atrial fibrillation"[All Fields] OR ("fibrillations"[All Fields] AND "auricular"[All Fields]) OR "fibrillations auricular"[All Fields]) OR ("atrial fibrillation"[MeSH Terms] OR ("atrial"[All Fields] AND "fibrillation"[All Fields]) OR "atrial fibrillation"[All Fields] OR ("persistent"[All Fields] AND "atrial"[All Fields] AND "fibrillation"[All Fields]) OR "persistent atrial fibrillation"[All Fields]) OR ("atrial fibrillation"[MeSH Terms] OR ("atrial"[All Fields] AND "fibrillation"[All Fields]) OR "atrial fibrillation"[All Fields] OR ("atrial"[All Fields] AND "fibrillation"[All Fields] AND "persistent"[All Fields]) OR "atrial fibrillation persistent"[All Fields]) OR ("atrial fibrillation"[MeSH Terms] OR ("atrial"[All Fields] AND "fibrillation"[All Fields]) OR "atrial fibrillation"[All Fields] OR ("atrial"[All Fields] AND "fibrillations"[All Fields] AND "persistent"[All Fields])) OR ("atrial fibrillation"[MeSH Terms] OR ("atrial"[All Fields] AND "fibrillation"[All Fields]) OR "atrial fibrillation"[All Fields] OR ("fibrillation"[All Fields] AND "persistent"[All Fields] AND "atrial"[All Fields]) OR "fibrillation persistent atrial"[All Fields]) OR

("atrial fibrillation"[MeSH Terms] OR ("atrial"[All Fields] AND "fibrillation"[All Fields]) OR "atrial fibrillation"[All Fields] OR ("fibrillations"[All Fields] AND "persistent"[All Fields] AND "atrial"[All Fields])) OR ("atrial fibrillation"[MeSH Terms] OR ("atrial"[All Fields] AND "fibrillation"[All Fields]) OR "atrial fibrillation"[All Fields] OR ("persistent"[All Fields] AND "atrial"[All Fields] AND "fibrillations"[All Fields]) OR "persistent atrial fibrillations"[All Fields]) OR ("atrial fibrillation"[MeSH Terms] OR ("atrial"[All Fields] AND "fibrillation"[All Fields]) OR "atrial fibrillation"[All Fields] OR ("familial"[All Fields] AND "atrial"[All Fields] AND "fibrillation"[All Fields]) OR "familial atrial fibrillation"[All Fields]) OR ("atrial fibrillation"[MeSH Terms] OR ("atrial"[All Fields] AND "fibrillation"[All Fields]) OR "atrial fibrillation"[All Fields] OR ("atrial"[All Fields] AND "fibrillation"[All Fields] AND "familial"[All Fields]) OR "atrial fibrillation familial"[All Fields]) OR ("atrial fibrillation"[MeSH Terms] OR ("atrial"[All Fields] AND "fibrillation"[All Fields]) OR "atrial fibrillation"[All Fields] OR ("atrial"[All Fields] AND "fibrillations"[All Fields] AND "familial"[All Fields])) OR ("atrial fibrillation"[MeSH Terms] OR ("atrial"[All Fields] AND "fibrillation"[All Fields]) OR "atrial fibrillation"[All Fields] OR ("familial"[All Fields] AND "atrial"[All Fields] AND "fibrillations"[All Fields])) OR ("atrial fibrillation"[MeSH Terms] OR ("atrial"[All Fields] AND "fibrillation"[All Fields]) OR "atrial fibrillation"[All Fields] OR ("fibrillation"[All Fields] AND "familial"[All Fields] AND "atrial"[All Fields])) OR ("atrial fibrillation"[MeSH Terms] OR ("atrial"[All Fields] AND "fibrillation"[All Fields]) OR "atrial fibrillation"[All Fields] OR ("fibrillations"[All Fields] AND "familial"[All Fields] AND "atrial"[All Fields])) OR ("atrial fibrillation"[MeSH Terms] OR ("atrial"[All Fields] AND "fibrillation"[All Fields]) OR "atrial fibrillation"[All Fields] OR ("paroxysmal"[All Fields] AND "atrial"[All Fields] AND "fibrillation"[All Fields]) OR "paroxysmal atrial fibrillation"[All Fields]) OR ("atrial fibrillation"[MeSH Terms] OR ("atrial"[All Fields] AND "fibrillation"[All Fields]) OR "atrial fibrillation"[All Fields] OR ("atrial"[All Fields] AND "fibrillation"[All Fields] AND "paroxysmal"[All Fields]) OR "atrial fibrillation paroxysmal"[All Fields]) OR ("atrial fibrillation"[MeSH Terms] OR ("atrial"[All Fields] AND "fibrillation"[All Fields]) OR "atrial fibrillation"[All Fields] OR ("atrial"[All Fields] AND "fibrillations"[All Fields] AND "paroxysmal"[All Fields]) OR "atrial fibrillations paroxysmal"[All Fields]) OR ("atrial fibrillation"[MeSH Terms] OR ("atrial"[All Fields] AND "fibrillation"[All Fields]) OR "atrial fibrillation"[All Fields] OR ("fibrillation"[All Fields] AND "paroxysmal"[All Fields] AND "atrial"[All Fields]) OR "fibrillation paroxysmal atrial"[All Fields]) OR ("atrial fibrillation"[MeSH Terms] OR ("atrial"[All Fields] AND "fibrillation"[All Fields]) OR "atrial fibrillation"[All Fields] OR ("fibrillations"[All Fields] AND "paroxysmal"[All Fields] AND "atrial"[All Fields]) OR "fibrillations paroxysmal atrial"[All Fields]) OR ("atrial fibrillation"[MeSH Terms] OR ("atrial"[All Fields] AND "fibrillation"[All Fields]) OR "atrial fibrillation"[All Fields] OR ("paroxysmal"[All Fields] AND "atrial"[All Fields] AND "fibrillations"[All Fields]) OR "paroxysmal atrial fibrillations"[All Fields]) OR ("atrial flutter"[MeSH Terms] OR ("atrial"[All Fields] AND "flutter"[All Fields]) OR "atrial flutter"[All Fields] OR ("atrial flutter"[MeSH Terms] OR ("atrial"[All Fields] AND "flutter"[All Fields]) OR "atrial flutter"[All Fields] OR ("atrial"[All Fields] AND "flutters"[All Fields]) OR "atrial flutters"[All Fields]) OR ("atrial flutter"[MeSH Terms] OR ("atrial"[All Fields] AND "flutter"[All Fields]) OR "atrial flutter"[All Fields] OR ("flutter"[All Fields] AND "atrial"[All Fields]) OR "flutter atrial"[All Fields]) OR ("atrial flutter"[MeSH Terms] OR ("atrial"[All Fields] AND "flutter"[All Fields]) OR "atrial flutter"[All Fields] OR ("flutters"[All Fields] AND "atrial"[All Fields]) OR "flutters atrial"[All Fields]) OR

("atrial flutter"[MeSH Terms] OR ("atrial"[All Fields] AND "flutter"[All Fields]) OR "atrial flutter"[All Fields] OR ("auricular"[All Fields] AND "flutter"[All Fields]) OR "auricular flutter"[All Fields]) OR ("atrial flutter"[MeSH Terms] OR ("atrial"[All Fields] AND "flutter"[All Fields]) OR "atrial flutter"[All Fields] OR ("auricular"[All Fields] AND "flutters"[All Fields]) OR "auricular flutters"[All Fields]) OR ("atrial flutter"[MeSH Terms] OR ("atrial"[All Fields] AND "flutter"[All Fields]) OR "atrial flutter"[All Fields] OR ("flutter"[All Fields] AND "auricular"[All Fields]) OR "flutter auricular"[All Fields]) OR ("atrial flutter"[MeSH Terms] OR ("atrial"[All Fields] AND "flutter"[All Fields]) OR "atrial flutter"[All Fields] OR ("flutters"[All Fields] AND "auricular"[All Fields]))) OR ("tachycardia, ventricular"[MeSH Terms] OR ("tachycardia"[All Fields] AND "ventricular"[All Fields]) OR "ventricular tachycardia"[All Fields] OR "tachycardia ventricular"[All Fields] OR ("tachycardia, ventricular"[MeSH Terms] OR ("tachycardia"[All Fields] AND "ventricular"[All Fields]) OR "ventricular tachycardia"[All Fields] OR ("ventricular"[All Fields] AND "tachycardias"[All Fields]) OR "ventricular tachycardias"[All Fields]) OR ("tachycardia, ventricular"[MeSH Terms] OR ("tachycardia"[All Fields] AND "ventricular"[All Fields]) OR "ventricular tachycardia"[All Fields] OR ("ventricular"[All Fields] AND "tachyarrhythmias"[All Fields]) OR "ventricular tachyarrhythmias"[All Fields]) OR ("tachycardia, ventricular"[MeSH Terms] OR ("tachycardia"[All Fields] AND "ventricular"[All Fields]) OR "ventricular tachycardia"[All Fields] OR ("tachyarrhythmia"[All Fields] AND "ventricular"[All Fields]) OR "tachyarrhythmia ventricular"[All Fields]) OR ("tachycardia, ventricular"[MeSH Terms] OR ("tachycardia"[All Fields] AND "ventricular"[All Fields]) OR "ventricular tachycardia"[All Fields] OR ("ventricular"[All Fields] AND "tachyarrhythmia"[All Fields]) OR "ventricular tachyarrhythmia"[All Fields]) OR ("tachycardia, ventricular"[MeSH Terms] OR ("tachycardia"[All Fields] AND "ventricular"[All Fields]) OR "ventricular tachycardia"[All Fields] OR ("ventricular"[All Fields] AND "tachycardia"[All Fields])) OR ("tachycardia, ventricular"[MeSH Terms] OR ("tachycardia"[All Fields] AND "ventricular"[All Fields]) OR "ventricular tachycardia"[All Fields] OR ("nonsustained"[All Fields] AND "ventricular"[All Fields] AND "tachycardia"[All Fields]) OR "nonsustained ventricular tachycardia"[All Fields]) OR ("tachycardia, ventricular"[MeSH Terms] OR ("tachycardia"[All Fields] AND "ventricular"[All Fields]) OR "ventricular tachycardia"[All Fields] OR ("nonsustained"[All Fields] AND "ventricular"[All Fields] AND "tachycardias"[All Fields]) OR "nonsustained ventricular tachycardias"[All Fields]) OR ("tachycardia, ventricular"[MeSH Terms] OR ("tachycardia"[All Fields] AND "ventricular"[All Fields]) OR "ventricular tachycardia"[All Fields] OR ("tachycardia"[All Fields] AND "nonsustained"[All Fields] AND "ventricular"[All Fields]) OR "tachycardia nonsustained ventricular"[All Fields]) OR ("tachycardia, ventricular"[MeSH Terms] OR ("tachycardia"[All Fields] AND "ventricular"[All Fields]) OR "ventricular tachycardia"[All Fields] OR ("ventricular"[All Fields] AND "tachycardia"[All Fields] AND "nonsustained"[All Fields]) OR "ventricular tachycardia nonsustained"[All Fields]) OR ("tachycardia, ventricular"[MeSH Terms] OR ("tachycardia"[All Fields] AND "ventricular"[All Fields]) OR "ventricular tachycardia"[All Fields] OR ("idiopathic"[All Fields] AND "ventricular"[All Fields] AND "tachycardia"[All Fields]) OR "idiopathic ventricular tachycardia"[All Fields]) OR ("tachycardia, ventricular"[MeSH Terms] OR ("tachycardia"[All Fields] AND "ventricular"[All Fields]) OR "ventricular tachycardia"[All Fields] OR ("idiopathic"[All Fields] AND "ventricular"[All Fields] AND "tachycardias"[All Fields]) OR "idiopathic ventricular tachycardias"[All Fields]) OR ("tachycardia, ventricular"[MeSH Terms] OR ("tachycardia"[All

Fields] AND "ventricular"[All Fields]) OR "ventricular tachycardia"[All Fields] OR ("tachycardia"[All Fields] AND "idiopathic"[All Fields] AND "ventricular"[All Fields]) OR "tachycardia idiopathic ventricular"[All Fields]) OR ("tachycardia, ventricular"[MeSH Terms] OR ("tachycardia"[All Fields] AND "ventricular"[All Fields]) OR "ventricular tachycardia"[All Fields] OR ("ventricular"[All Fields] AND "tachycardia"[All Fields] AND "idiopathic"[All Fields]) OR "ventricular tachycardia idiopathic"[All Fields])) OR ("ventricular fibrillation"[MeSH Terms] OR ("ventricular"[All Fields] AND "fibrillation"[All Fields]) OR "ventricular fibrillation"[All Fields] OR ("ventricular fibrillation"[MeSH Terms] OR ("ventricular"[All Fields] AND "fibrillation"[All Fields]) OR "ventricular fibrillation"[All Fields] OR ("fibrillation"[All Fields] AND "ventricular"[All Fields]) OR "fibrillation ventricular"[All Fields]) OR ("ventricular fibrillation"[MeSH Terms] OR ("ventricular"[All Fields] AND "fibrillation"[All Fields]) OR "ventricular fibrillation"[All Fields] OR ("fibrillations"[All Fields] AND "ventricular"[All Fields]) OR "fibrillations ventricular"[All Fields]) OR ("ventricular fibrillation"[MeSH Terms] OR ("ventricular"[All Fields] AND "fibrillation"[All Fields]) OR "ventricular fibrillation"[All Fields] OR ("ventricular"[All Fields] AND "fibrillations"[All Fields]) OR "ventricular fibrillations"[All Fields])) OR (("paranasal sinuses"[MeSH Terms] OR ("paranasal"[All Fields] AND "sinuses"[All Fields]) OR "paranasal sinuses"[All Fields] OR "sinus"[All Fields] OR "sinus s"[All Fields]) AND ("bradycardia"[MeSH Terms] OR "bradycardia"[All Fields] OR "bradycardias"[All Fields]))))

Search Strategy of Embase

#40. #33 AND #39

#39. #34 OR #35 OR #36 OR #37 OR #38

#38. 'sinus bradycardia'

#37. 'ventricular fibrillation' OR 'fibrillation, ventricular' OR 'fibrillations, ventricular' OR 'ventricular fibrillations'

#36. 'tachycardia, ventricular' OR 'ventricular tachycardias' OR 'ventricular tachyarrhythmias' OR 'tachyarrhythmia, ventricular' OR 'ventricular tachyarrhythmia' OR 'ventricular tachycardia' OR 'nonsustained ventricular tachycardia' OR 'nonsustained ventricular tachycardias' OR 'tachycardia, nonsustained ventricular' OR 'ventricular tachycardia, nonsustained' OR 'idiopathic ventricular tachycardia' OR 'idiopathic ventricular tachycardias' OR 'tachycardia, idiopathic ventricular' OR 'ventricular tachycardia,diopathic'

#35. 'atrial flutter' OR 'atrial flutters' OR 'flutter, atrial' OR 'flutters, atrial' OR 'auricular flutter' OR 'auricular flutters' OR 'flutter, auricular' OR 'flutters, auricular'

#34. 'atrial fibrillation' OR 'trial fibrillations' OR 'fibrillation, atrial' OR 'fibrillations, atrial' OR 'auricular fibrillation' OR 'auricular fibrillations' OR 'fibrillation, auricular' OR 'fibrillations, auricular' OR 'persistent atrial fibrillation' OR 'atrial fibrillation, persistent' OR 'atrial fibrillations, persistent' OR 'fibrillation, persistent atrial' OR 'fibrillations, persistent atrial' OR 'persistent atrial fibrillations' OR 'familial atrial fibrillation' OR 'atrial fibrillation, familial' OR 'atrial fibrillations, familial' OR 'familial atrial fibrillations' OR 'fibrillation, familial atrial' OR 'fibrillations, familial atrial' OR 'paroxysmal atrial fibrillation' OR 'atrial fibrillation, paroxysmal' OR 'atrial fibrillations, paroxysmal' OR 'fibrillation, paroxysmal atrial' OR 'fibrillations, paroxysmal atrial' OR 'paroxysmal atrial fibrillations'

#33. #1 OR #2 OR #3 OR #4 OR #5 OR #6 OR #7 OR #8 OR #9 OR #10 OR #11 OR #12 OR #13 OR #14 OR #15 OR #16 OR #17 OR #18 OR #19 OR #20 OR #21 OR #22 OR #23 OR #24 OR

#25 OR #26 OR #27 OR #28 OR #29 OR #30 OR #31 OR #32
#32. 'lik066':ti,ab
#31. 'licogliflozin':ti,ab
#30. 'lx4211':ti,ab
#29. 'sotagliflozin':ti,ab
#28. 'remogliflozin':ti,ab
#27. 'ipragliflozin':ti,ab
#26. 'sergliflozin':ti,ab
#25. 'pf04971729':ti,ab
#24. 'ertugliflozin':ti,ab
#23. 'ts071':ti,ab
#22. 'luseogliflozin':ti,ab
#21. 'tofogliflozin':ti,ab
#20. 'ta7284':ti,ab
#19. 'canagliflozin':ti,ab
#18. 'bi 10773':ti,ab
#17. 'empagliflozin':ti,ab
#16. 'bms 512148':ti,ab
#15. 'dapagliflozin':ti,ab
#14. 'gliflozin':ti,ab
#13. 'gliflozins':ti,ab
#12. 'inhibitor, sglt-2':ti,ab
#11. 'inhibitor, sglt2':ti,ab
#10. 'sglt 2 inhibitor':ti,ab
#9. 'sglt-2 inhibitor':ti,ab
#8. 'sglt2 inhibitor':ti,ab
#7. 'sglt2 inhibitors':ti,ab
#6. 'sglt 2 inhibitors':ti,ab
#5. 'sglt-2 inhibitors':ti,ab
#4. 'sodium glucose transporter 2 inhibitor':ti,ab
#3. 'sodium-glucose transporter 2 inhibitor':ti,ab
#2. 'sodium glucose transporter 2 inhibitors':ti,ab
#1. 'sodium glucose cotransporter 2 inhibitor'/exp

Search Strategy of Web of Science

1: TS=(lik066 OR licogliflozin OR lx4211 OR sotagliflozin OR remogliflozin OR ipragliflozin OR sergliflozin OR pf04971729 OR ertugliflozin OR ts071 OR luseogliflozin OR tofogliflozin OR ta7284 OR canagliflozin OR bi 10773 OR empagliflozin OR bms 512148 OR dapagliflozin OR gliflozin OR gliflozins OR inhibitor, sglt-2 OR inhibitor, sglt2 OR sglt 2 inhibitor OR sglt-2 inhibitor OR sglt2 inhibitor OR sglt2 inhibitors OR sglt 2 inhibitors OR sglt-2 inhibitors OR sodium glucose transporter 2 inhibitor OR sodium-glucose transporter 2 inhibitor OR sodium glucose transporter 2 inhibitors OR sodium glucose cotransporter 2 inhibitor) and Preprint Citation Index (Exclude – Database)

2: TS=(atrial fibrillation OR trial fibrillations OR fibrillation, atrial OR fibrillations, atrial OR

auricular fibrillation OR auricular fibrillations OR fibrillation, auricular OR fibrillations, auricular OR persistent atrial fibrillation OR atrial fibrillation, persistent OR atrial fibrillations, persistent OR fibrillation, persistent atrial OR fibrillations, persistent atrial OR persistent atrial fibrillations OR familial atrial fibrillation OR atrial fibrillation, familial OR atrial fibrillations, familial OR familial atrial fibrillations OR fibrillation, familial atrial OR fibrillations, familial atrial OR paroxysmal atrial fibrillation OR atrial fibrillation, paroxysmal OR atrial fibrillations, paroxysmal OR fibrillation, paroxysmal atrial OR fibrillations, paroxysmal atrial OR paroxysmal atrial fibrillations) and Preprint Citation Index (Exclude – Database)

3: TS=(atrial flutter OR atrial flutters OR flutter, atrial OR flutters, atrial OR auricular flutter OR auricular flutters OR flutter, auricular OR flutters, auricular) and Preprint Citation Index (Exclude – Database)

4: TS=(tachycardia, ventricular OR ventricular tachycardias OR ventricular tachyarrhythmias OR tachyarrhythmia, ventricular OR ventricular tachyarrhythmia OR ventricular tachycardia OR nonsustained ventricular tachycardia OR nonsustained ventricular tachycardias OR tachycardia, nonsustained ventricular OR ventricular tachycardia, nonsustained OR idiopathic ventricular tachycardia OR idiopathic ventricular tachycardias OR tachycardia, idiopathic ventricular OR ventricular tachycardia, idiopathic) and Preprint Citation Index (Exclude – Database)

5: TS=(ventricular fibrillation OR fibrillation, ventricular OR fibrillations, ventricular OR ventricular fibrillations) and Preprint Citation Index (Exclude – Database)

6: TS=(sinus bradycardia) and Preprint Citation Index (Exclude – Database)

7: #6 OR #5 OR #4 OR #3 OR #2 and Preprint Citation Index (Exclude – Database)

8: #7 AND #1 and Preprint Citation Index (Exclude – Database)
